# Proteomic Analysis Is Needed to Understand the Vulnerability of *Bunodosoma cavernatum* Sea Anemones to Climate Change

**DOI:** 10.1021/acs.jproteome.4c00780

**Published:** 2025-01-27

**Authors:** Mayra P. Becerra-Amezcua, Fernando M. Matadamas-Guzmán, Lorena Hernández-Orihuela, Isabel Guerrero-Legarreta, Xochitl Guzmán-García

**Affiliations:** a Department of Hydrobiology, Division of Biological and Health Sciences, Ecotoxicology Laboratory, Universidad Autónoma Metropolitana, Iztapalapa Unit, Mexico City C. P. 09340, Mexico; b Instituto de Biotecnología, Universidad Nacional Autónoma México, Avenida Universidad 2001, Chamilpa, Cuernavaca, Morelos C. P. 62210, Mexico; c Department of Biotechnology, Division of Biological and Health Sciences, Macromolecules Laboratory, Universidad Autónoma Metropolitana, Iztapalapa Unit, Mexico City C. P. 09340, Mexico; d Postgraduate in Energy and Environment, Basic Sciences and Engineering Division, Universidad Autónoma Metropolitana, Iztapalapa Unit, Mexico City C. P. 09340, Mexico

**Keywords:** proteomic, *Bunodosoma*, oxygen stress, thermal stress, climate change

## Abstract

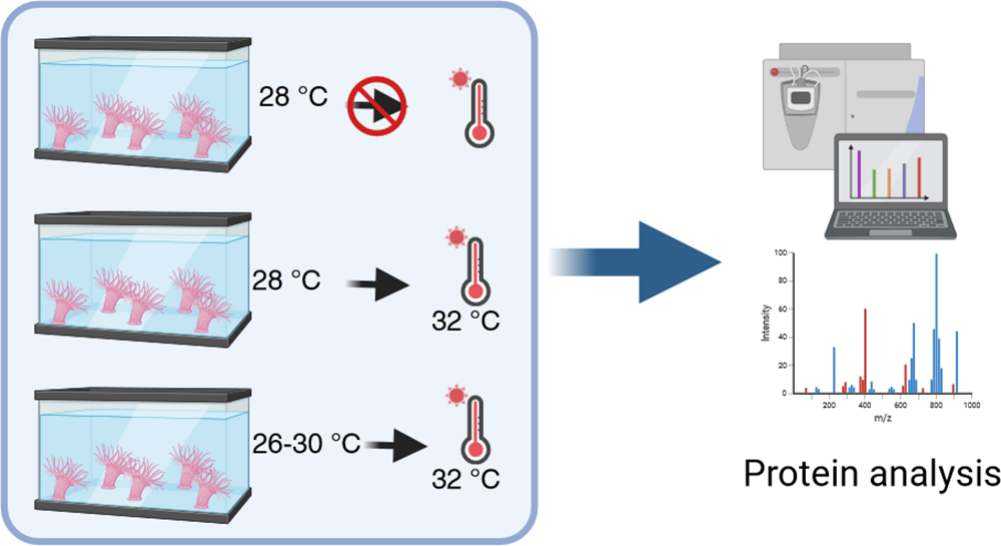

Sea anemones play a crucial role in marine ecosystems. Recent studies have highlighted their physiological and ecological responses to thermal stress. Therefore, our objective was to perform a proteomic analysis of *Bunodosoma cavernatum* sea anemones in the Gulf of Mexico, subjected to thermal stress, to understand whether these organisms activate specific processes to resist increased temperature. We submitted one group of sea anemones to variable temperatures (26 to 32 °C) and another group to a constant temperature (28 °C) for 1.5 months. Then we subjected them to thermal stress (32 °C) for 2 weeks. We evaluated the enzymatic activity and proteome in the columns and tentacles. The main effect of the temperature regime change is a reduction in mass. Also, sea anemones synthesized proteins related to the activation of the immune system and protection against temperature. We observed decreased peroxidase activity, while superoxide dismutase activity was higher only in the constant temperature group. On the basis of these data, we deduce that *B. cavernatum* sea anemones are vulnerable to climate change because they stop producing toxins in their tentacles when faced with thermal stress and activate cellular responses that make them susceptible to pathogens. These responses are not sufficient to guarantee an optimal health state.

## Introduction

Climate change drives the rise in ocean temperatures, which affects marine organisms, including sea anemones. These cnidarians play a crucial role in marine ecosystems due to their symbiotic relationships with photosynthetic algae and their ability to act as bioindicators of environmental changes. Sea anemones exhibit notable physiological changes when exposed to elevated temperatures; for example, high temperatures can induce bleaching in anemones that harbor symbiotic algae, mainly due to heat-induced respiratory dysfunction.^1,2^ In addition, researchers have documented that thermal stress affects the microbiome of sea anemones, affecting their overall health and symbiotic relationships.^3^ In general, published studies have provided valuable information about the responses of sea anemones to thermal stress. However, the key proteins that facilitate adaptation and resilience mechanisms in sea anemones have not yet been characterized.

Proteomics, the large-scale study of proteins, offers a powerful approach to understanding the molecular responses of sea anemones to environmental stressors. Proteomic characterization helps to elucidate the mechanisms underlying the anemone responses to thermal stress. Heat shock proteins (HSPs), for example, play a critical role in helping anemones cope with elevated temperatures by maintaining cellular homeostasis and facilitating protein folding.^4,5^ Recent proteomic studies have shown that sea anemones can upregulate specific HSPs in response to temperature increases, allowing them to survive in changing environments.^6^ Furthermore, proteomic analyses have revealed complex interactions between sea anemones and pathogens, showing that thermal stress can modulate immune responses and pathogen virulence.^7^ Sea anemones exhibit several mechanisms to cope with thermal stress, but the details remain unclear.

Understanding the adaptive molecular mechanisms that organisms employ is essential for developing effective conservation strategies to protect marine ecosystems in the face of global climate change. Therefore, we performed proteomic analysis of the *Bunodosoma cavernatum* sea anemones in the Gulf of Mexico to understand how these organisms cope with thermal stress after exposure to constant or variable temperatures.

## Experimental Section

### Obtention of Organisms and Identification of Species

Sea anemones were developed incidentally in PIMVS-CIDMIRA-UAMI (Center of Wildlife Management Research and Teaching for Comprehensive Management of Aquatic Organisms, SEMARNAT-UAMI permit 09-LR0900-11-17) through a collection of organisms carried out in the rocky intertidal zone at a depth of approximately 0.5 to 3 m in the Tecolutla region, Veracruz, Mexico. Subsequently, the organisms were transferred to the Ecotoxicology Laboratory of the Universidad Autónoma Metropolitana, Iztapalapa Unit, following the ethical recommendations of the University, lowering the temperature of the transfer water (filtered seawater) by 2 °C every 15 min to avoid metabolic stress. When they reached the laboratory, the organisms were acclimatized for admission to 40 L artificial seawater fish tanks (Instant Ocean) with controlled temperature and salinity (28 °C and 32 ppm), placing five organisms per fish tank. The organisms were kept under controlled lighting conditions with cycles of 12 h of light and 12 h of darkness (Aqueon, mod. 100121106) and were fed daily dry food (Polyp lab, Reef-Roids) and newly hatched brine shrimp.

The species was identified through morphological aspects described by González-Muñoz et al. (2013),^8^ considering the oral disc diameter, number of tentacles, coloration, marginal projections, cnidoma, internal anatomy through histology, etc.

### Temperature Bioassay

The *B. cavernatum* sea anemones (*n* = 30) were obtained from Tecolutla, Veracruz, Mexico. They weighed 6.94 ± 1.28 g and measured 2.3 ± 0.1 cm in diameter and 1.9 ± 0.15 cm in height. The collected sea anemones were placed in three different fish tanks with a salinity of 32 ppt. In fish tanks 1 and 2, the sea anemones were kept at a constant temperature (28 °C), while in fish tank 3, the sea anemones were subjected to a variable temperature regime (raising 2 °C every 8 h, from 26 to 30 °C in 24 h) for 1.5 months. Subsequently, only sea anemones in fish tanks 2 and 3 were subjected to thermal stress (32 °C) for 2 weeks; fish tank 1 was used as a control (experiment based on ref (9)).

### Protein Isolation

At the end of the experiment, sea anemones were anesthetized by decreasing the water temperature (1 °C every 15 min) until they no longer responded to tactile stimuli (approximately 5 °C). Tentacle biopsies and the middle of the column were collected by using a dermal perforator (5 mm in diameter). The tissue was frozen in liquid nitrogen. The protein was extracted with a glass homogenizer using a lysis buffer (0.5 M NaCl, 100 mM Tris and 10 mM EDTA, pH 7.5). After homogenization, the samples were centrifuged at 14,000 rpm for 20 min at 4 °C (Velocity 14R, 6627001, Dynamica, UK). The supernatant was removed and stored at −20 °C until use.

### Enzymatic Assays

The activity of two enzymes—peroxidase and superoxide dismutase—was determined in the supernatants of the column and tentacle. Both enzymes are involved in response to oxidative stress. Peroxidase breaks down hydrogen peroxide (H_2_O_2_) into water and oxygen. The MAK092-1KT Peroxidase Activity Assay Kit (Sigma-Aldrich, USA) was used to determine the peroxidase assay, 50 μL of each sample to be tested was placed, and the enzyme activity was calculated as follows: at the beginning of the assay, the absorbance was measured at 570 nm; after 30 min, it was measured again on a Multiskan plate reader (Thermo Scientific, 51119200) to calculate the change in absorbance (Δ*A*). The Δ*A* was applied to the H_2_O_2_ standard curve to get *B* nmol of H_2_O_2_ generated by peroxidase in the given reaction time.
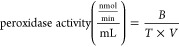
where *B* = amount of H_2_O_2_ from the standard curve (in nmol), *T* = incubation time (minutes), and *V* = the volume of the sample added to the reaction well (mL).

SOD catalyzes the dismutation of the superoxide anion (O_2_^–^); it is one of the most important antioxidant enzymes. The SOD Determination Kit 19160 (Sigma-Aldrich; USA) was used to determine SOD activity according to the manufacturer’s instructions, which were as follows: add 20 μL of each sample and blank 2 well and add 20 μL of ultrapure H_2_O to each blank 1 and blank 3 well, incubate the plate at 37 °C for 20 min, read the absorbance at 450 nm using a microplate reader, and calculate the SOD activity (inhibition rate %) using the following equation:
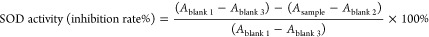


The result was divided by the weight of protein added to obtain SOD activity/weight of protein.

### Sample Preparation for Liquid Chromatography Tandem Mass Spectrometry (LC–MS/MS)

After protein extraction (see above), the protein concentration was determined using the Bradford method,^10^ with bovine serum albumin as the standard. From each sample, 50 μg of total protein was subjected to shotgun proteomic profiling following the same workflow. First, each sample was reduced with 10 mM dithiothreitol (DTT) for 30 min at 60 °C, alkylated with 55 mM iodoacetamide (IAA; Sigma-Aldrich, USA), and subjected to in solution digestion with trypsin (Promega, Madison, WI, USA) to obtain peptides. The tryptic peptides were desalted using a Ziptip C18 (Millipore, USA) and concentrated in a Savant SPD1010 SpeedVac (Thermo Fisher Scientific, USA). The desalted and concentrated peptides were resuspended in 20 uL of Milli-Q water for quantification.

### LC–MS/MS Analysis

An LC–MS system comprising an Ultimate 3000 Dionex–LTQ Orbitrap Velos (Thermo Fisher Scientific, USA) and an Acclaim PEPMAP C18 capillary column (Thermo Fisher Scientific, USA) was used to analyze tryptic peptides generated by digestion in a solution of each sample. Prior to performing the experiment, the LTQ Orbitrap Velos instrument was calibrated by using a mixed ion positive ion calibration solution (LTQ ESI, Pierce, Thermo Fisher Scientific, USA). For analysis, 0.5 μg of tryptic peptides was injected into the system. The peptides were separated using a gradient of 4 to 85% solvent B (solvent A was water and solvent B was acetonitrile, both with 0.1% formic acid) for 120 min, maintaining a flow rate of 300 nL/min. All spectra were acquired in positive-ion mode. The acquisition method involved a dynamic exclusion set to a maximum of 500 ions and 70 s for the exclusion duration. Full-scan MS spectra from *m*/*z* 400 to 1600 were acquired at a resolving power of 60,000, a 3.0 Da isolation width, and 35 arbitrary normalized collision energy units. Collision-induced dissociation and high-energy collision-activated dissociation were alternately used for fragmentation.

### Bioinformatic Analysis of the MS Data

Each raw data file was subjected to a search in Proteome Discoverer 1.4 to identify proteins. This search used the UniProt Actiniaria database, a fragment tolerance of 0.60 Da, a parent tolerance of 20 ppm, a fixed modification (C), and variable modifications of deamidation (N, Q) and oxidation (M).

### Statistical Analysis

One-way analysis of variance (ANOVA) was used to determine whether there were significant differences between the weight groups using Šídák’s multiple comparison test. A two-way ANOVA was used to determine whether there were significant differences between the SOD activity and H_2_O_2_ activity groups using Dunnett’s multiple comparison test. The GraphPad Prism 10.2.3 software (GraphPad Software, San Diego, CA, USA) was used for this analysis.

## Results

**Figure 1:**
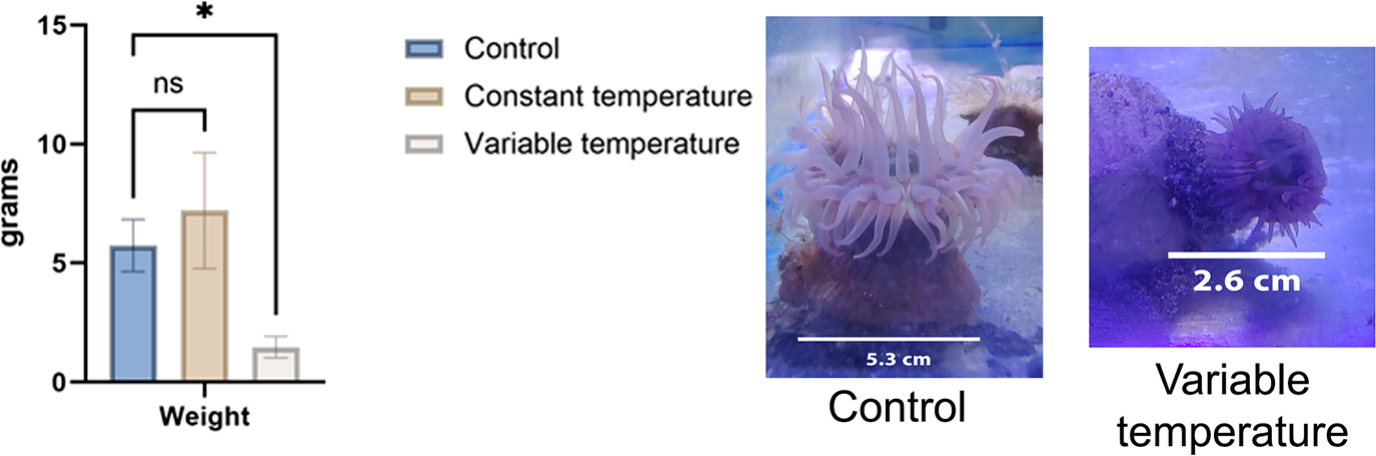
Mass of the *B. cavernatum* sea anemones at the end of the experiment.

The most notable observation was that sea anemones subjected to variable temperature showed a marked and significant decrease in mass (wet weight) compared to the control group (Figure 1).

### Enzymatic Activity

**Figure 2:**
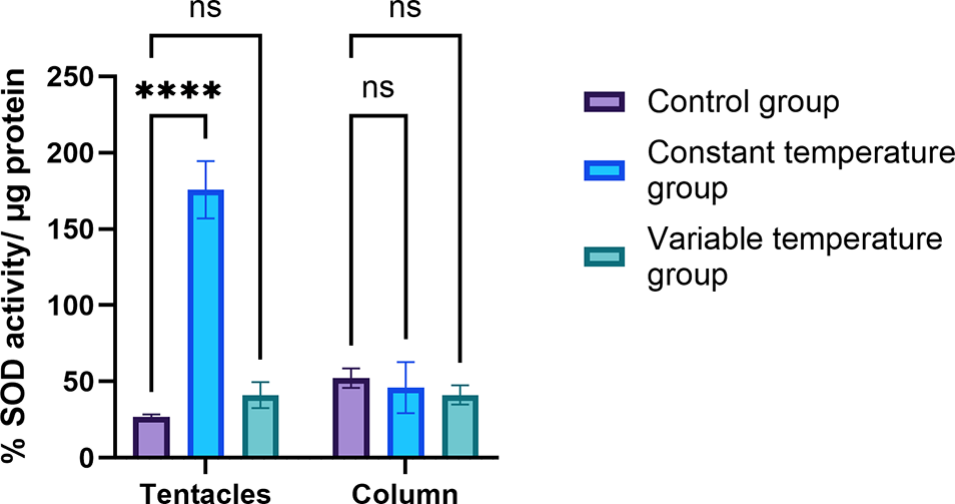
Superoxide dismutase (SOD) activity in the column and tentacles of *B. cavernatum* sea anemones. ****, with statistically representative differences; ns, without statistically representative differences.

Our enzyme analyses revealed that peroxidase was present only in the column of the control group with 0.17 ± 0.01 nmol/min/mL activity. On the other hand, we detected SOD in the column and tentacles of all three groups. The only significant difference was an increase in tentacles in the constant temperature group compared to the control group (Figure 2).

### Proteomic Analysis

We processed proteins obtained from the sea anemone column and tentacles by using LC–MS (Table S1). We observed different protein patterns depending on the temperature group. We manually inspected the data by decreasing the restriction of the number of unique peptides identified by protein sequence to 1. However, we considered only cases where a single peptide showed high coverage of the identified protein (Table S2).

**Figure 3:**
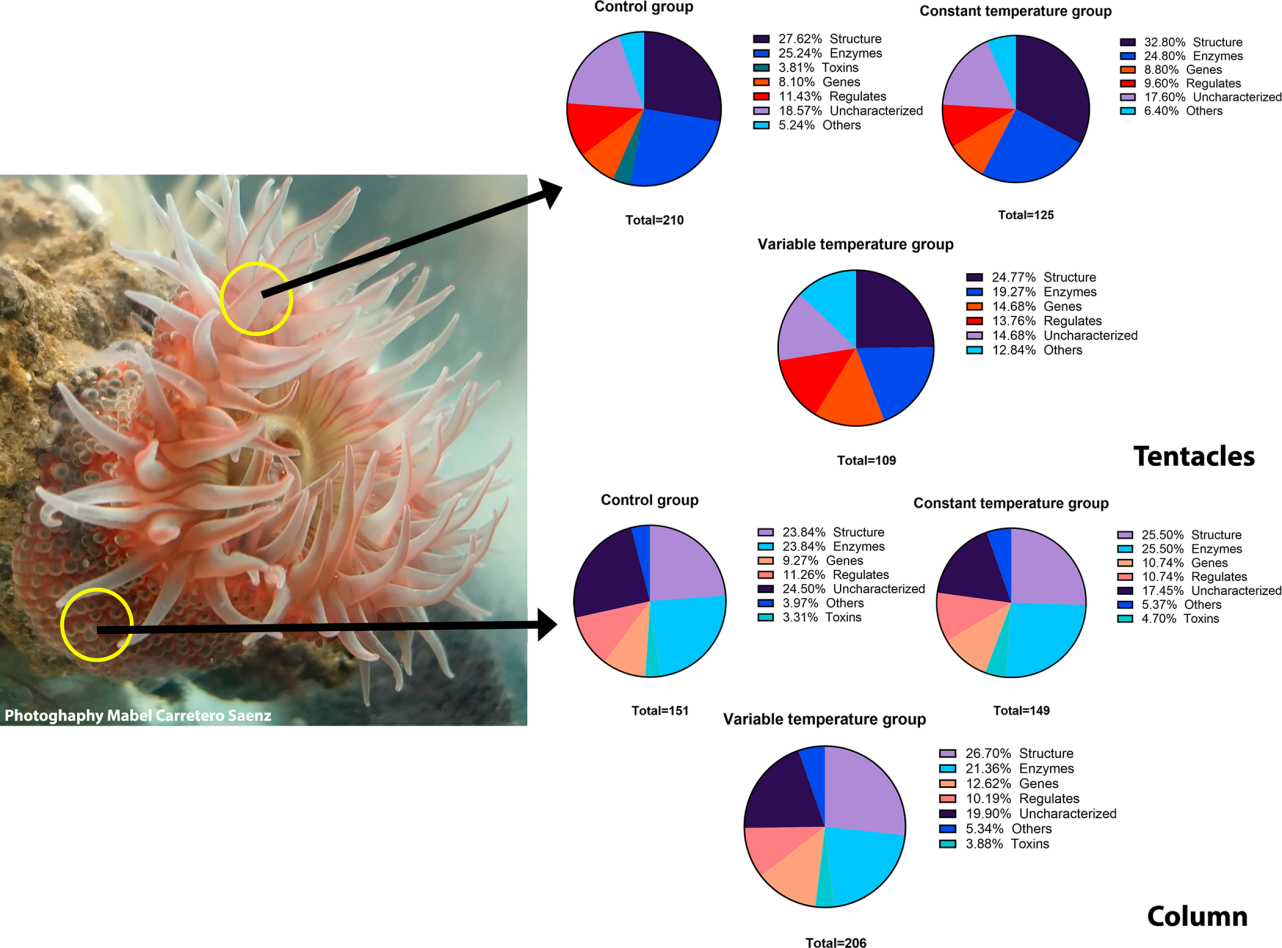
Tentacle and column protein profiles of the *B. cavernatum* sea anemones based on proteomics. Photograph of sea anemone courtesy of Ariadna Mabel Carretero Saenz, 2025.

In the tentacles, we identified 210 proteins in the control group, 125 proteins in the constant temperature group, and 109 proteins in the variable temperature group. Spectrin with a molecular weight of 280 kDa was present in the constant and variable temperature groups but not in the control group. The abundance of alpha-actin-1-like was significantly higher than that of the 101 kDa control. The failed axon connection protein (45 kDa) was present in all three groups. We found the translationally controlled tumor protein homologue (22 kDa) only in the variable temperature group. Trefoil factor 2-like isoform X1 (20 kDa) was present in the control and constant temperature groups but not in the variable temperature group. It is noteworthy that the groups subjected to thermal stress for 2 weeks did not present toxins in their tentacles (Figure 3).

In the column, we identified 151 proteins in the control group, 149 in the constant temperature group, and 206 in the variable temperature group (Figure 3). One of the most abundant proteins in the column was actin (42 kDa).

Most of the identified proteins are structural and located in the cytoplasm, cytoskeleton, and cytosol. Several of these proteins are enzymes or toxins and regulate processes related to gene expression, bioluminescence, and reproduction, among others.

#### Tentacles

**Figure 4:**
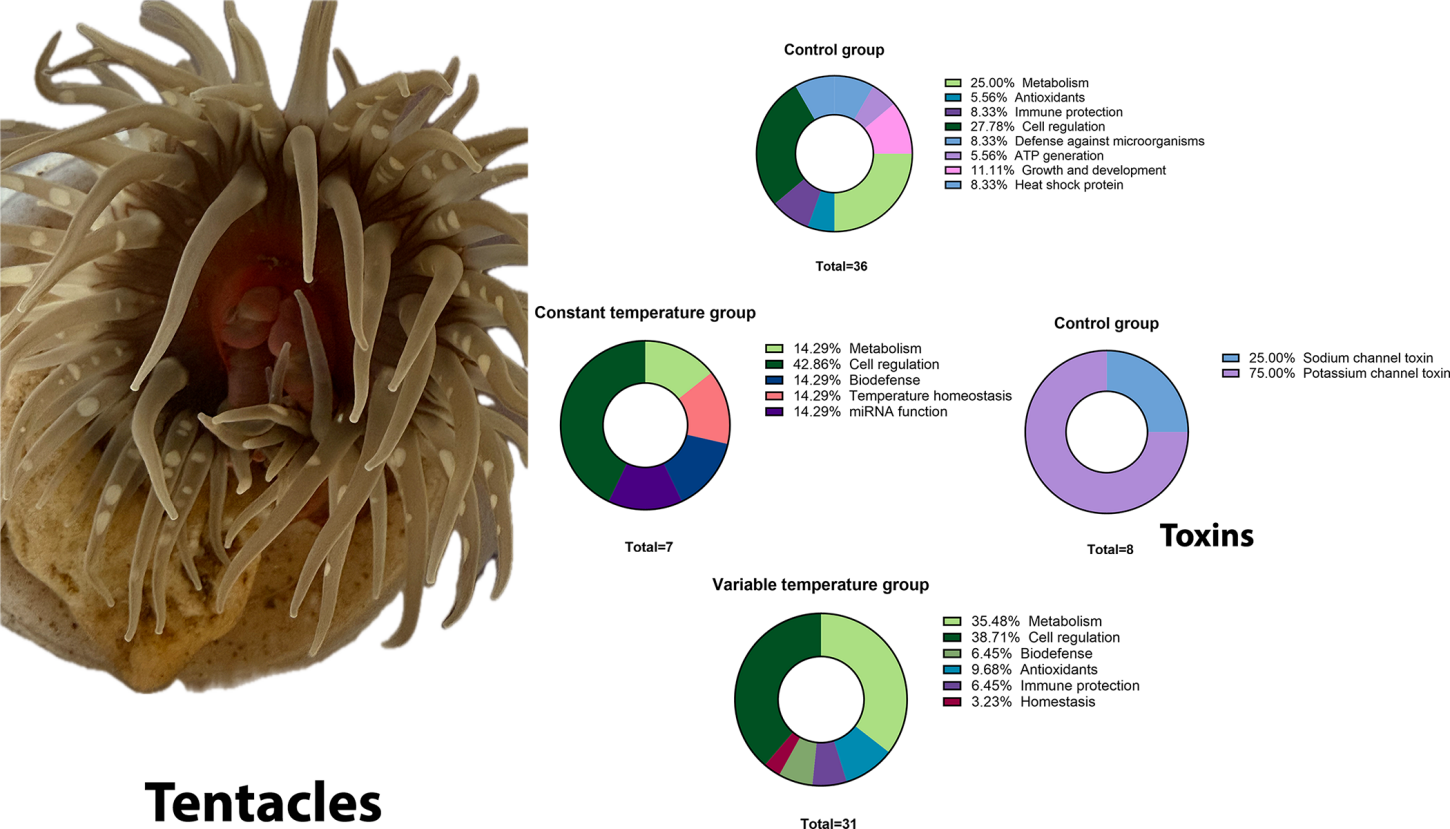
Tentacle protein function of *B. cavernatum* sea anemones.

In the control group, we identified several toxins in the tentacles. Sea anemones synthesize these toxins to incapacitate their prey and defend themselves against predators. We identified several toxins, namely, sushi, von Willebrand factor type A, epidermal growth factor (EGF), 1-like pentraxin domain-containing protein, and Toxin Bcg III 31.16 (fragment). We also identified proteins involved in the defense against microorganisms, especially proteins involved in immune protection, endocytosis, and cell regulation. When subjected to temperature stress, the regulatory protein profile in the tentacles differed between the constant and variable temperature groups (Figure 4). In the constant temperature group, we identified proteins involved in temperature homeostasis and inflammation. In the variable temperature group, we observed proteins that activate the immune response, glucose metabolism, and some responses to oxidative stress (Figure 4).

In Figure 4, we can observe proteins present only in the analyzed group; unlike the experimental groups, the control group presents 36 proteins not found in the experimental groups, whose most representative function is cellular regulation, followed by proteins involved in metabolism, growth, and development, proteins involved in immune protection, and heat shock proteins, among others. In the constant temperature group, we can find 7 proteins that are not found in the other groups, whose primary function is cellular regulation, and in the variable temperature group, we can find 31 proteins not found in the other groups, whose primary function is cellular regulation and metabolism, it should be noted that, in this group, the presence of proteins with antioxidant activity increased. In addition to those reported above, toxins with a function in sodium and potassium channels are found only in the control group (Tables S3–S5).

#### Column

**Figure 5:**
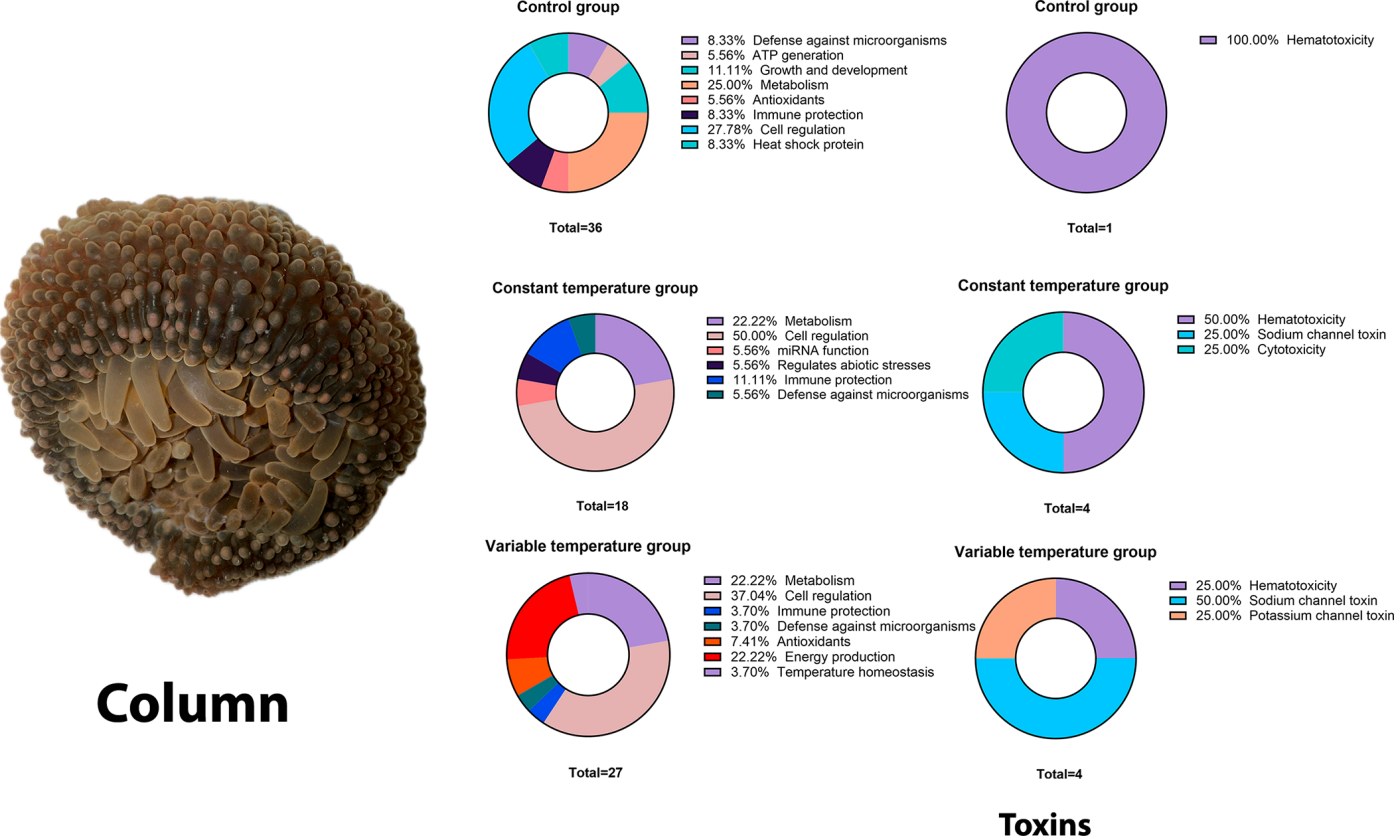
Column protein function of the *B. cavernatum* sea anemones.

In the control group column, we identified proteins related to muscle assembly; inflammation; energy production; photosynthesis; protein, lipid, and carbohydrate metabolism; sushi toxins, von Willebrand factor type A, EGF, and pentraxin domain-containing protein 1-like; and regulatory proteins related to neurogenesis, inflammation, muscle migration, and cell proliferation. In the constant temperature group, we observed proteins related to abiotic stressors and activation of the immune system against pathogens such as bacteria; 18 proteins were identified only in this group, 50% of which have a function in cellular regulation, followed by proteins with metabolic function. Unlike the control group, we identified other toxins such as metalloendopeptidases and Bcg III, as well as regulatory proteins related to the maintenance of plasticity in the development of synapses and cell proliferation; 36 proteins were identified that were not found at other experimental times. The majority fulfill the function of cellular regulation followed by metabolism and the growth and development of organisms. In the variable temperature group, we identified proteins related to protection against oxidative stress, protection against bacteria, detoxification, generation of antioxidants, energy production related to glycogenesis, and temperature homeostasis, and 27 proteins appeared only in this group. As in the previous one, the main functions of the proteins are cellular regulation and metabolism. However, unlike the groups described above, proteins related to energy production appeared in this one. This group also produced different toxins compared to the other two groups (kappa-actitoxin-Bcs4a and U-actitoxin-Bcs2a) and regulatory proteins related to homeostasis and the immune system (Figure 5).

Regarding the function of the toxins present in the column, we can observe that, in all groups, we find toxins with hematotoxicity; in the group with constant temperature, toxins with cytotoxicity and toxins with activity in sodium channels appear, and in the group with variable temperature, above all, they have toxins with function in sodium and potassium channels (Tables S6–S8).

## Discussion

The effects of ocean warming on the physiology of marine organisms have been reported. However, there have only been a few studies on the effects of ocean warming on sea anemones.^11^ Moreover, proteomic analyses have focused more on describing proteins related to basic physiological processes.^12−15^ In this study, we described the physiological effects of temperature stress on *B. cavernatum* sea anemones in the face of climate change.

Researchers have suggested that corals, which live in environments with natural temperature variability, experience less bleaching when they are exposed to thermal stress. In other words, exposure to variable temperatures could make them more resistant to thermal stress.^9^ We observed certain differences between sea anemones subjected to constant and variable temperatures. Consistent with other species of sea anemones, *B. cavernatum* subjected to temperature variations, including exposure to high temperatures, lost body mass. Indeed, sea anemones cannot balance their metabolic needs with energy input, and thus, their biomass is reduced when exposed to high temperatures.^16^

An increase in the water temperature affects some marine invertebrates; for example, corals may undergo irreversible bleaching. As a result of this increase in temperature, marine organisms can be subjected to oxidative stress due to the high level of formation of reactive oxygen species (ROS). While low levels of ROS play crucial roles in normal cell metabolism and protection against pathogens, high levels could be lethal in some cases.^17^ Of the two antioxidant enzymes examined, SOD was present in both the column and tentacles of all organisms. However, the only significant change in SOD activity was an increase in the constant temperature group, specifically in the tentacles. According to Richier et al. (2005),^18^ anemones with symbiotic organisms show differences in their SOD response compared to anemones that do not have symbiotic organisms. *B. cavernatum* has been reported to support zooxanthellae, which are single-celled dinoflagellates.^8^ This symbiosis may explain the increase in SOD activity in the tentacles of the constant temperature group. Furthermore, sea anemones subjected to thermal stress lose their symbiont organisms, especially when they are subjected to thermal stress for several days.^19^ This loss of symbiont organisms could explain why SOD activity did not increase in the tentacles of the variable temperature group.

Peroxidases are related to the innate immune response in sea anemones; however, they have received little attention.^20^ High peroxidase activity confers resistance to infection. The control group presented only peroxidase activity in the column. This activity decreased significantly in the column of the constant and variable temperature groups; the data suggest that exposure to thermal stress makes sea anemones more vulnerable to infection.

The tentacles of sea anemones produce toxins that allow these organisms to capture prey and defend against predators.^21^ Furthermore, tentacles contain proteins with other functions, including transport, metabolism, signaling, and vesicular trafficking, among others.^12^ We identified proteins with various functions in the tentacles. Among these, structural proteins, enzymes, toxins, proteins related to genetic processes, and regulators, among others, stand out. Although the protein profile was different for each group, most of the proteins we identified are structural proteins and enzymes. We found that sea anemones subjected to heat stress lost the ability to produce toxins in their tentacles; however, they can generate toxins in the column; these toxins replace the function of the toxins produced in the tentacles. This finding is novel: There is little information on the effect of temperature on cnidarian toxins.^13,22,23^ Hence, we have provided evidence that climate change affects toxin production in sea anemones.

Unlike the tentacles, in the column, we identified more proteins in the variable temperature group compared with the control and constant temperature groups. Unique proteins we found in the variable temperature group include antioxidant proteins, proteins related to temperature homeostasis and the immune system, and the toxins kappa-antitoxin-Bsc4a, a neurotoxin that blocks voltage-activated potassium channels,^24^ and U-antitoxin-Bcs2a, which has similar activity and can mediate hemolysis.^14^ These changes indicate that the column nematocysts produce more toxins to compensate for the reduced synthesis of toxins when exposed to thermal stress.

The proteins we identified in this study coincide with other proteomics studies that involve anemones.^7,15,25^ These studies proposed using caspases as biomarkers of climate change. However, based on our results, we propose that HSPs, peroxiredoxin, adenosylhomocysteinase, and EF-hand domain-containing proteins are biomarkers.

Although some species have been shown to become resistant when subjected to a variable temperature regime,^9^ we found that this is not the case for *B. cavernatum* sea anemones. Although there was a clear difference in the physiological processes of sea anemones subjected to constant and variable temperatures, in both cases, the immune defense processes were activated. What is evident is that while sea anemones subjected to constant temperature synthesize proteins related to temperature homeostasis and activation of the immune system, sea anemones subjected to variable temperature synthesize proteins related to oxidative stress. This may be because, as noted in a previous study, there is a loss of important zooxanthella after 21 days of heat stress.^26^ Oxidative stress acts at the molecular level during bleaching, causing apoptosis under heat stress. However, enzyme antioxidant defenses are induced. Additional ROS production causes a significant increase in cellular damage,^27^ but this depends on the species and the ability of its exogenous antioxidants to mitigate the effects of heat stress.^28^ These differences indicate that the variable temperature group lost its zooxanthellae during the experiment, which made them more vulnerable to 2 week thermal stress.

## Conclusions

Our results show that the physiological processes of the *B. cavernatum* sea anemones change under thermal stress conditions. When subjected to thermal stress, they lose biomass and increase the synthesis of proteins related to homeostasis. In addition, they lose the ability to synthesize toxins in their tentacles and exhibit a reduction in the activity of an important antioxidant enzyme, namely, peroxidase. The health of *B. cavernatum* sea anemones appears to be closely related to their symbiont organisms.

## Data Availability

Mass spectrometry proteomics data have been deposited in the MassIVE archive via the Center for Computational Mass Spectrometry partner repository with the data set identifier MassIVE MSV000095911. doi:10.25345/C5CJ87 × 8B

## Supporting Information Available

Sample report (Table S1) (XLSX)
Peptide report (Table S2) (XLSX)
Main enzymes identified in the tentacles (Table S3); main toxins identified in the tentacles (Table S4); main regulatory proteins identified in the tentacles (Table S5); main enzymes identified in the column (Table S6); main toxins identified in the column (Table S7); and main regulatory proteins identified in the column (Table S8) (PDF)
