# Supplementary material for: Proteomic Analysis Is Needed to Understand the Vulnerability of Bunodosoma cavernatum Sea Anemones to Climate Change
Source: J Proteome Res. 2025 Jan 27;24(2):742–9. doi: 10.1021/acs.jproteome.4c00780 (PMC11811985; doi:10.1021/acs.jproteome.4c00780)
Supplement: Supplementary file 3 — pr4c00780_si_003.pdf [file pr4c00780_si_003.pdf]

Supporting information for publication

Title: Proteomic Analysis is needed to understand the vulnerability of *Bunodosoma cavernatum* sea anemones to climate change.

Mayra P. Becerra-Amezcua[a,d]; Fernando M. Matadamas-Guzmán[a,d]; Lorena Hernández-Orihuela[b];. Isabel Guerrero-Legarreta[c]; Xochitl Guzmán-García[a]

a Department of Hydrobiology, Division of Biological and Health Sciences, Ecotoxicology Laboratory, Universidad Autónoma Metropolitana, Iztapalapa Unit, Mexico City, Mexico, C.P. 09340, mapabeam@xanum.uam.mx, cbi2213801080@izt.uam.mx, xgg@xanum.uam.mx

b Instituto de Biotecnología, Universidad Nacional Autónoma México, Avenida Universidad 2001, Chamilpa, Cuernavaca, Mor., Mexico, C.P. 62210, lorena.hernandez@ibt.unam.mx

c Department of Biotechnology, Division of Biological and Health Sciences, Macromolecules Laboratory, Universidad Autónoma Metropolitana, Iztapalapa Unit, Mexico City, Mexico, C.P. 09340, meat@xanum.uam.mx

d Postgraduate in Energy and Environment, Basic Sciences and Engineering Division, Universidad Autónoma Metropolitana, Iztapalapa Unit, Mexico City, Mexico, C.P. 09340

Table of contents:

Table S1 - Sample report (xlsx)

Table S2 - Peptide report (xlsx)

Table S3 - Main enzymes identified in the tentacles (doc)

Table S4 - Main toxins identified in the tentacles (doc)

Table S5 - Main regulatory proteins identified in the tentacles (doc)

Table S6 - Main enzymes identified in the column (doc)

Table S7 - Main toxins identified in the column (doc)

Table S8 - Main regulatory proteins identified in the column (doc)

Supporting Information: Function of the proteins identified in the different experimental groups

Table S3. The main enzymes identified in the tentacles of the *Bunodosoma cavernatum* sea anemone groups based on proteomic analysis.

**Control group**					
**Accession number**	**Identified protein**	**Organism**	**% coverage**	**Function**	**Reference**
A0A6P8HMR8	Lysozyme-like	*Actinia tenebrosa*	6.2	Defense against bacteria	[1]
A0A6P8IWN4	Cathepsin L1-like	*Actinia tenebrosa*	8	Plays essential biological roles in innate immunity and the development of animals	[2]
A0A913Y784	Aldehyde dehydrogenase domain-containing protein	*Exaiptasia diaphana*	5.1	Participates in detoxification, biosynthesis, antioxidant, and regulatory functions	[3]
A0A6P8HVF3	Cathepsin B-like	*Actinia tenebrosa*	5.1	Play multiple roles in the life cycle of parasites, such as food uptake, immune invasion, and pathogenesis	[4]
A0A6P8IV21	ATP synthase subunit beta	*Actinia tenebrosa*	4.5	ATP generation	[5]
A0A6P8HH62	Retinal dehydrogenase 1-like	*Actinia tenebrosa*	5.1	Controls growth and development	[6]
A0A913X1G2	ATP synthase subunit alpha	*Exaiptasia diaphana*	1.8	ATP generation	[5]
A0A6P8IFP3	Catalase	*Actinia tenebrosa*	6.3	An essential enzyme that protects the cell from oxidative damage caused by reactive oxygen species	[7]
A0A6P8HHY2	Peptidyl-prolyl cis-trans isomerase	*Actinia tenebrosa*	5.5	A variety of biological functions, ranging from cell cycle regulation to bacterial infection	[8]
A0A6P8I4X0	Adenosylhomocysteinase	*Actinia tenebrosa*	2.8	Embryonic development; cellular stress; and DNA, RNA, and histone methylation	[9]
A0A6P8I3A7	Cathepsin X	*Actinia tenebrosa*	4.7	Regulates migration, adhesion, proliferation, and maturation in immune and neuronal cells, along	[10]

				with phagocytosis and signal transduction	
A7S5Z0	Phosphopyruvate hydratase	*Nematostella vectensis*	3.9	Glycolytic function and plays a variety of roles in pathophysiological settings, including oncogenesis, tumor progression, ischemia, and bacterial infection	[11]
A0A6P8J1E9	Cytosolic non-specific dipeptidase-like isoform X1	*Actinia tenebrosa*	3.8	Protein metabolism	[12]
A0A6P8ITN1	PC3-like endoprotease variant B	*Actinia tenebrosa*	1.9	Protein metabolism	[13]
A0A6P8I7L6	Protein disulfide-isomerase	*Actinia tenebrosa*	12	Protein metabolism	[14]
A0A6P8HEJ7	Peptidylprolyl isomerase	*Actinia tenebrosa*	10	Roles in the folding of newly synthesized proteins and the function of the immune system	[15]
A0A6P8HTG6	Calpain-B-like isoform X1	*Actinia tenebrosa*	1.9	Involved in various physiological processes such as cell proliferation, cell migration, cell cycle progression, apoptosis, cytoskeletal remodeling, and signal transduction	[16]
A7S0B5	Methylcrotonoyl-CoA carboxylase	*Nematostella vectensis*	1.7	Prevents the accumulation of toxic intermediaries, facilitates the generation of branched-chain fatty acids, and/or provides energy to the cell	[17]
A7S2S9	Protein kinase domain-containing protein	*Nematostella vectensis*	0.59	Protein metabolism	[18]
A0A6P8HK35	dihydrofolate reductase	*Actinia tenebrosa*	7.5	Cell proliferation and growth	[19]
A0A6P8IV09	Protein disulfide isomerase-like 2-2	*Actinia tenebrosa*	3.6	Protein metabolism	[20]
A0A913XFF8	Aminotransferase class I/class II domain-containing protein	*Exaiptasia diaphana*	3.3	Involved in amino acid transport, which is important for carbon and nitrogen supply; pH homeostasis; osmoprotection; virulence; detoxification; signal transduction; and ion gradient generation	[21]
A0A913WS37	AB hydrolase-1 domain-containing protein	*Exaiptasia diaphana*	3,0	Energy metabolism, cell signaling, growth, and development	[22]
A0A6P8HKB1	Dihydropyrimidine dehydrogenase [NADP(+)]	*Actinia tenebrosa*	0.88	Pyrimidine metabolism	[23]
A0A913XLB6	Acyl-coenzyme A synthetase ACSM3, mitochondrial-like	*Exaiptasia diaphana*	1.5	Lipid metabolism	[24]

Constant temperature group					
**Accession number**	**Identified protein**	**Organism**	**% coverage**	**Function**	**Reference**
A0A913X1F1	Cytosol aminopeptidase domain-containing protein	*Exaiptasia diaphana*	10	Protein metabolism	[25]
A7RXU0	Staphylococcal nuclease domain-containing protein	*Nematostella vectensis*	1.1	Plays an important role in miRNA function	[26]
A0A6P8HV19	Very long-chain specific acyl-CoA dehydrogenase, mitochondrial-like	*Actinia tenebrosa*	2.0	Temperature homeostasis	[27]
A7S9S7	Thioesterase domain-containing protein	*Nematostella vectensis*	6.4	Biodefense	[28]
**Variable temperature group**					
**Accession Number**	**Identified proteins**	**Organism**	**% coverage**	**Function**	**Reference**
A0A6P8IF06	Beta-N-acetylhexosaminidase	*Actinia tenebrosa*	0.56	Sphingolipid catabolism	[29]
A0A6P8HS46	S-adenosylmethionine-dependent nucleotide dehydratase RSAD2	*Actinia tenebrosa*	6.9	Innate immune response to viruses	[30]
A0A6P8IWJ0	1-Phosphatidylinositol-3-phosphate 5-kinase	*Actinia tenebrosa*	0.76	Plays an important role in the maintenance of endomembrane homeostasis, including endocytosis, vacuole formation, and vacuolar acidification processes	[31]
A0A6P8IPS6	S-formylglutathione hydrolase	*Actinia tenebrosa*	5.7	Formaldehyde metabolic process	[32]
A7S417	Phosphoenolpyruvate carboxykinase (GTP)	*Nematostella vectensis*	2.2	Produces glucose from pyruvate derived from amino acid metabolism	[33]
A0A6P8HEL0	Malate dehydrogenase	*Actinia tenebrosa*	11	Gluconeogenesis	[34]
A0A6P8HYS1	Non-specific serine/threonine protein kinase	*Actinia tenebrosa*	0.46	Regulates cell proliferation, programmed cell death (apoptosis), cell differentiation, and embryonic development	[35]
A0A913Y0V7	RING-type domain-containing protein	*Exaiptasia diaphana*	0.54	Protein metabolism	[36]
A0A913YFD9	E3 ubiquitin-protein ligase HERC1	*Exaiptasia diaphana*	0.26	Regulates cell migration	[37]
A0A6P8ICC2	Protein phosphatase 1 regulatory subunit 7-like	*Actinia tenebrosa*	2.5	Putative tumor suppressor via dephosphorylation-mediated inactivation of AKT and mitogen-activated protein kinase signaling pathways	[38]
A0A913X2P9	Histidine ammonia-lyase	*Exaiptasia diaphana*	3.1	Protein metabolism	[39]
A7S394	Aminopeptidase (fragment)	*Nematostella vectensis*	1.4	Protein metabolism	[40]
A0A913XCU7	Apple domain-containing protein	*Exaiptasia diaphana*	5.2	Protein metabolism	[41]
A0A6P8HYM7	Isoaspartyl peptidase/L-asparaginase-like	*Actinia tenebrosa*	5.5	Protein metabolism	[42]

A0A913WXT4	E3 ubiquitin-protein ligase RNF213-like	*Exaiptasia diaphana*	1.2	Acts as a key immune sensor by catalyzing lipid ubiquitination	[43]
A0A6P8J044	Glutathione transferase	*Actinia tenebrosa*	5.5	Plays a critical role in cellular detoxification against xenobiotics and noxious compounds, and protection against oxidative stress	[44]
A7RR23	SGNH hydrolase-type esterase domain-containing protein	*Nematostella vectensis*	7.9	Carbohydrate metabolism	[45]
A0A6P8IEC7	Alanine transaminase	*Actinia tenebrosa*	3.3	Intermediary metabolism of glucose	[46]

Table S4. The main toxins identified in the tentacles of the *B. cavernatum* sea anemone control group based on proteomic analysis.

**Control group**			
**Accession number**	**Identified protein**	**Organism**	**% coverage**
A0A0S1M175	Sodium channel toxin protein (fragment)	*Anemonia sulcata*	84
P86468	Toxin Bcs III 15.09 (fragment)	*Bunodosoma caissarum*	44
P86461	Toxin Bcg III 31.16 (fragment)	*Bunodosoma cangicum*	87
P86462	Toxin Bcg III 28.78 (fragment)	*Bunodosoma cangicum*	49
C0HJC4	Kappa-actitoxin-Bcs4a	*Bunodosoma caissarum*	24
P84919	U-actitoxin-Bcs2a	*Bunodosoma caissarum*	71
P86862	KappaPI-actitoxin-Ael3a	*Anthopleura elegantissima*	14
A0A0S1M1A1	Type III potassium channel toxin protein (fragment)	*Anemonia sulcata*	15

Table S5. The main regulatory proteins identified in the tentacles of the *B. cavernatum* sea anemone groups based on proteomic analysis.

**Control group**					
**Accession number**	**Identified protein**	**Organism**	**% coverage**	**Function**	**Reference**
A0A6P8HFH9	Heat shock cognate 71 kDa protein	*Actinia tenebrosa*	11	Endocytosis	[47]
A7T144	Heat shock protein 70	*Nematostella vectensis*	5.8	Endocytosis	[47]
A0A6P8IYN5	Cysteine and glycine-rich protein 1-like	*Actinia tenebrosa*	18	Gene transcription, cell movement, and cell proliferation	[48]
A0A6P8IAZ2	Endoplasmic reticulum chaperone BiP-like	*Actinia tenebrosa*	5.8	Protein metabolism	[49]
A0A6P8HEJ6	Protein D2-like	*Actinia tenebrosa*	12	Regulates a full spectrum of physiological processes, including movement, sleep,	[50]

				memory, and attention	
A0A913WRN9	Neurexin-4	*Exaiptasia diaphana*	2	Synaptic maintenance and plasticity	51
A0A913XEK1	BRICHOS domain-containing protein	*Exaiptasia diaphana*	3.9	Regulates cell proliferation	52
A0A913YLM6	Dentin sialophosphoprotein-like	*Exaiptasia diaphana*	0.32	Cell differentiation	53
A0A913X7I1	Son of sevenless homolog 1	*Exaiptasia diaphana*	0.94	Regulates cell proliferation	54
A7SDG9	G-protein coupled receptors family 3 profile domain-containing protein	*Nematostella vectensis*	0.98	Transduction of extracellular stimuli into intracellular signals	55
A0A6P8H835	Signal transducer and activator of transcription	*Actinia tenebrosa*	2.1	Regulates the expression of genes involved in cell survival, proliferation, differentiation, development, the immune response, and, among other essential biological functions, hematopoiesis	56
A0A6P8HUR1	Rho GDP-dissociation inhibitor 1-like	*Actinia tenebrosa*	5.1	In glioma cells, inhibits cell migration and invasion	57
A0A6P8HQ76	Calumenin-like	*Actinia tenebrosa*	2.6	Alleviates endoplasmic reticulum stress, which can lead to cell death	58
A0A913XWB1	10 kDa heat shock protein, mitochondrial	*Exaiptasia diaphana*	9.8	Prevents misfolding and promotes the refolding and proper assembly of unfolded polypeptides generated under stress conditions in the mitochondrial matrix	59
A7SV03	Ubiquitin-like domain-containing protein	*Nematostella vectensis*	1.1	Autophagy, protein trafficking, inflammation and immune responses, transcription, DNA repair, RNA splicing, and cellular differentiation	60
**Constant temperature group**					
**Accession number**	**Identified protein**	**Organism**	**% coverage**	**Function**	**Reference**
A0A6P8J6M5	Calmodulin-like protein 12	*Actinia tenebrosa*	3.1	Controls diverse cellular functions by regulating the activity of various target proteins	61
A0A913X949	Calreticulin	*Exaiptasia diaphana*	4.9	Cell adhesion, gene expression, and RNA stability	62
A0A6P8IVY9	Calmodulin-like	*Actinia tenebrosa*	7.1	Mediates crucial processes such as inflammation, metabolism, apoptosis, smooth	63

				muscle contraction, intracellular movement, short- and long-term memory, and the immune response	
**Variable temperature group**					
**Accession number**	**Identified protein**	**Organism**	**% coverage**	**Function**	**Reference**
A0A913Y5S4	G-protein coupled receptors family 1 profile domain-containing protein	*Exaiptasia diaphana*	2.3	Cellular response to hormone stimulus	[63]
A0A913XFQ2	Roc domain-containing protein	*Exaiptasia diaphana*	0.95	Regulates protein function and signaling pathways	[64]
A0A913X903	FERM domain-containing protein	*Exaiptasia diaphana*	4.4	Links actin filaments to adhesion proteins	[65]
A0A913YCZ3	Thioredoxin domain-containing protein	*Exaiptasia diaphana*	8.1	Plays a role in protection against oxidative stress in mitochondria	[66]
A0A6P8IQI0	Radixin-like	*Actinia tenebrosa*	6.4	A membrane–cytoskeletal crosslinker in actin-rich cell surface structures; thus, thought to be essential for cortical cytoskeleton organization, cell motility, adhesion, and proliferation	[67]
A0A6P8IWK4	14-3-3-like protein	*Actinia tenebrosa*	3.8	Cell cycle progression, DNA damage response, programmed cell death, cytoskeletal dynamics, and transcriptional control of gene expression	[68]
A0A6P8I5D4	Lethal(2) giant larvae protein homolog 1-like isoform X1	*Actinia tenebrosa*	1.3	Proliferation, differentiation, and tissue organization of neuroepithelial cells	[69]
A0A913YCZ3	Thioredoxin domain-containing protein	*Nematostella vectensis*	8.1	Plays roles in protection against oxidative stress in mitochondria	[66]
A0A913Y035	RAD50-interacting protein 1-like	*Exaiptasia diaphana*	1.2	Cellular processes, including DNA double-strand break repair, cell cycle checkpoint activation, telomere maintenance, and meiosis	[70]
A7RR10	Rubicon homology domain-containing protein (Fragment)	*Nematostella vectensis*	3.4	Modulates the inflammatory response and viral replication	[71]
A7SEC9	DIX domain-containing protein (Fragment)	*Nematostella vectensis*	3.4	Protein–protein interaction	[72]
A0A6P8J5R1	Arp2/3 complex 34 kDa subunit	*Actinia tenebrosa*	7.7	Regulates actin polymerization and, together with an	[73]

				activating nucleation-promoting factor, mediates the formation of a branched actin network	
A0A6P8IIN4	Rab GDP dissociation inhibitor	*Actinia tenebrosa*	4.3	Regulates the Rab small G proteins implicated in neurotransmission	[74]

Table S6. The main enzymes identified in the column of the *B. cavernatum* sea anemone groups based on proteomic analysis.

Control group					
**Accession number**	**Identified protein**	**Organism**	**% coverage**	**Function**	**Reference**
A0A6P8IKJ4	Titin homolog	*Actinia tenebrosa*	0.25	A key component in the assembly and functioning of muscles In non-muscle cells, it seems to play a role in chromosome condensation and chromosome segregation during mitosis	[75]
A7S165	1-phosphatidylinositol 4-kinase	*Nematostella vectensis*	1.1	Defines the membranes of the Golgi and trans-Golgi network and regulates trafficking to and from the Golgi	[76]
A0A6P8H3L0	Chitinase-3-like protein 1	*Actinia tenebrosa*	2.5	Mediates inflammation, macrophage polarization, apoptosis, and carcinogenesis	[77]
A8DX89	Glyceraldehyde-3-phosphate dehydrogenase (Fragment)	*Urticina eques*	9.4	Production of energy and photosynthesis	[78]
A0A6P8J0J2	Acidic endochitinase-like	*Actinia tenebrosa*	3.5	Inhibits chitin-induced innate inflammation and augments chitin-free, allergen-induced Th2 inflammation	[79]
A0A913YLV7	Sphingolipid 4-desaturase	*Exaiptasia diaphana*	0.54	Involved in cell cycle control	[80]
A7S6G4	Lipase	*Nematostella vectensis*	3.0	Breaks down triglycerides into free fatty acids and glycerol	[81]
A0A6P8IWJ0	1-Phosphatidylinositol-3-phosphate 5-kinase	*Actinia tenebrosa*	1.2	Participates in the phosphatidylinositol signaling system and regulation of the actin cytoskeleton	[82]
A0A913XMR0	Peptidase S9 prolyl oligopeptidase catalytic domain-containing protein	*Exaiptasia diaphana*	1.5	Protein metabolism	[83]

A0A6P8I4N6	Ectonucleoside triphosphate diphosphohydrolase 4-like	*Actinia tenebrosa*	2.6	Pyrimidine metabolism	[84]
A0A6P8H8F7	Carboxypeptidase Q	*Actinia tenebrosa*	4.2	Protein metabolism	[85]
A0A6P8IBI1	Beta-galactosidase-1-like protein 2 isoform X2	*Actinia tenebrosa*	2.0	Carbohydrate metabolism	[86]
A0A6P8I9I0	Alanine--glyoxylate aminotransferase	*Exaiptasia diaphana*	3.3	Protein metabolism	[87]
A0A6P8HE66	inorganic diphosphatase	*Actinia tenebrosa*	4.9	Essential for cell growth	[88]
**Constant temperature group**					
**Accession number**	**Identified protein**	**Organism**	**% coverage**	**Function**	**Reference**
A0A6P8I2F3	Protein LONG AFTER FAR-RED 3-like	*Actinia tenebrosa*	1.9	Regulates abiotic stresses	[89]
A0A913YJQ2	GH18 domain-containing protein	*Exaiptasia diaphana*	1.0	Carbohydrate metabolism	[90]
A0A6P8HVF3	Cathepsin B-like	*Actinia tenebrosa*	5.1	Plays multiple roles in the life cycle of parasites, such as food uptake, immune invasion, and pathogenesis	[4]
A7SSJ4	Gamma-glutamyltransferase (fragment)	*Nematostella vectensis*	3.3	Protein metabolism	[91]
A0A6P8HH62	Retinal dehydrogenase 1-like	*Actinia tenebrosa*	2.7	Controls growth and development	[6]
A0A913X8N6	Fatty acid desaturase domain-containing protein	*Exaiptasia diaphana*	2.5	Lipid metabolism	[92]
A0A6P8HHY2	Peptidyl-prolyl cis-trans isomerase	*Actinia tenebrosa*	8.5	A variety of biological functions, ranging from cell cycle regulation to bacterial infection	[8]
A7RXU0	Staphylococcal nuclease domain-containing protein	*Nematostella vectensis*	1.1	Plays an important role in miRNA function	[26]
A0A6P8IL95	dual-specificity kinase	*Actinia tenebrosa*	1.5	Various cellular functions by downregulating the mitogen-activated protein kinase cascade	[93]
A0A6P8HWM9	Phosphatidylglycerophosphatase and protein-tyrosine phosphatase 1-like	*Actinia tenebrosa*	7.3	May prevent intrinsic apoptosis, probably by regulating mitochondrial membrane integrity	[94]
A7RYA6	Separase	*Nematostella vectensis*	0.77	Chromosome segregation, DNA damage repair, spindle elongation, and centrosome replication	[95]
A7SGN5	Cathepsin X (fragment)	*Nematostella vectensis*	3.2	Regulates immune and neuronal cell migration, adhesion, proliferation, and maturation, together with phagocytosis and signal transduction	[96]
**Variable temperature group**					
**Accession number**	**Identified protein**	**Organism**	**% coverage**	**Function**	**Reference**
A7RK73	Thioredoxin domain-containing protein	*Nematostella vectensis*	16	Plays roles in protection against oxidative stress in the mitochondria	[66]

A0A6P8I1G4	Phosphopyruvate hydratase	*Actinia tenebrosa*	5.0	Glycolytic function and plays a variety of roles in pathophysiological settings, including oncogenesis, tumor progression, ischemia, and bacterial infection	11
A0A913Y784	Aldehyde dehydrogenase domain-containing protein	*Exaiptasia diaphana*	3.1	Participates in detoxification, biosynthesis, antioxidant, and regulatory functions	3
A0A6P8IH34	Creatine kinase B-type-like	*Actinia tenebrosa*	12	Plays a *central role in energy transduction in tissues* with large, fluctuating energy demands, such as skeletal muscle, heart, and brain	97
A0A6P8IV21	ATP synthase subunit beta	*Actinia tenebrosa*	5.8	ATP generation	5
A0A6P8IPY6	Phosphoenolpyruvate carboxykinase (GTP)	*Actinia tenebrosa*	5.1	Produces glucose from pyruvate derived from amino acid metabolism	33
A0A913WWP6	Glyceraldehyde-3-phosphate dehydrogenase	*Exaiptasia diaphana*	2.4	Energy production and photosynthesis	78
A0A913X1G2	ATP synthase subunit alpha	*Exaiptasia diaphana*	5.6	ATP generation	5
A0A913XVX4	Creatine kinase	*Exaiptasia diaphana*	5.3	Plays a *central role in energy transduction in tissues* with large, fluctuating energy demands, such as skeletal muscle, heart, and brain	97
A0A913XNB5	Choline/carnitine acyltransferase domain-containing protein	*Exaiptasia diaphana*	1.7	Lipid metabolism	98
A7S418	Phosphoenolpyruvate carboxykinase (GTP)	*Nematostella vectensis*	1.9	Produces glucose from pyruvate derived from amino acid metabolism	33
A0A6P8ITU3	Tyrosine-protein phosphatase non-receptor type 23-like isoform X1	*Actinia tenebrosa*	0.70	Plays a role in ciliogenesis	99
A0A6P8HQ83	cAMP and cAMP-inhibited cGMP 3',5'-cyclic phosphodiesterase 10A-like	*Actinia tenebrosa*	3.6	May play a critical role in regulating cAMP and cGMP levels in the striatum, a region of the brain that contributes to the control of movement and cognition	100
A0A6P8HEL0	Malate dehydrogenase	*Actinia tenebrosa*	2.4	Gluconeogenesis	34
A0A6P8HV19	Very long-chain specific acyl-CoA dehydrogenase, mitochondrial-like	*Actinia tenebrosa*	2.7	Temperature homeostasis	27
A0A913YTX4	Alpha-1,3-mannosyl-glycoprotein 2-beta-N-acetylglucosaminyltransferase	*Exaiptasia diaphana*	1.3	Carbohydrate metabolism	101
A0A6P8IFK1	Alanine--tRNA ligase	*Actinia tenebrosa*	1.4	Plays a major role in rescue of stalled ribosomes via trans-translation.	102

A7RER2	Arginine kinase 1	*Nematostella vectensis*	4.3	Protein metabolism	[103]
A0A6P8J3L1	Polyamine oxidase 1-like	*Actinia tenebrosa*	3.4	Protein metabolism	[104]

Table S7. The main toxins identified in the column of the *B. cavernatum* sea anemone groups based on proteomic analysis.

Control group			
**Accession number**	**Identified protein(s)**	**Organism**	**% coverage**
A7SNM7	Sushi, von Willebrand factor type A, epidermal growth factor, and pentraxin domain-containing protein 1-like	*Nematostella vectensis*	0.72
**Constant temperature group**			
**Accession number**	**Identified protein**	**Organism**	**% coverage**
A0A913XES3	von Willebrand factor A domain-containing protein 5B1	*Exaiptasia diaphana*	0.85
A7RL03	Metalloendopeptidase (fragment)	*Nematostella vectensis*	5.8
P86469	Toxin Bcg III 15.67 (fragment)	*Bunodosoma cangicum*	76
A0A2Z2DZ35	Actinoporin (Fragment)	*Bunodosoma cavernatum*	15
**Variable temperature group**			
**Accession number**	**Identified protein**	**Organism**	**% coverage**
P86462	Toxin Bcg III 28.78 (fragment)	*Bunodosoma cangicum*	24
C0HJC4	Kappa-actitoxin-Bcs4a	*Bunodosoma caissarum*	24
P84919	U-actitoxin-Bcs2a	*Bunodosoma caissarum*	22
A7S0V4	Metalloendopeptidase	*Nematostella vectensis*	5.8

Table S8. The main regulatory proteins identified in the column of the *B. cavernatum* sea anemone groups based on proteomic analysis.

Control group					
**Accession number**	**Identified protein**	**Organism**	**% coverage**	**Function**	**Reference**
A0A6P8JG91	Bone morphogenetic protein 7-like	*Actinia tenebrosa*	5.3	Plays an important role in various biological processes, including embryogenesis, hematopoiesis, neurogenesis, and skeletal morphogenesis	[105]
A7RYE8	F-box only protein 3	*Nematostella vectensis*	3.2	Acts as a regulator of inflammation by mediating ubiquitination	[106]
A0A913YWJ2	G-protein coupled receptors family 2 profile 2 domain-containing protein	*Exaiptasia diaphana*	0.40	Transduction of extracellular stimuli into	[55]

				intracellular signals	
A0A6P8ICF6	Dynamin-binding protein	*Actinia tenebrosa*	0.91	Promotes directional migration of vascular smooth muscle cells	[107]
A0A913XPR3	Protein spindly	*Exaiptasia diaphana*	2.1	Plays a role in cell migration	[108]
A0A913YAW4	Neural proliferation differentiation and control protein 1	*Exaiptasia diaphana*	4.4	Correlated with the regulation of cellular proliferation and differentiation	[109]
A0A6P8J187	MORC family CW-type zinc finger protein 3-like	*Actinia tenebrosa*	2.8	The major immediate early promoter in myeloid cells	[110]
**Constant temperature group**					
**Accession number**	**Identified protein**	**Organism**	**% coverage**	**Function**	**Reference**
A0A6P8IAZ2	Endoplasmic reticulum chaperone BiP-like	*Actinia tenebrosa*	1.6	Protein metabolism	[49]
A0A6P8HEJ6	Protein D2-like	*Actinia tenebrosa*	12	Regulates a spectrum of physiological processes, including movement, sleep, memory, and attention	[50]
A0A913WRN9	Neurexin-4	*Exaiptasia diaphana*	0.94	Synaptic maintenance and plasticity	[51]
A0A6P8IQR8	Heat shock protein beta-6-like	*Actinia tenebrosa*	8.1	Vasodilation, platelet function, and insulin resistance in smooth and cardiac muscle	[111]
A7RWJ1	Arf-GAP domain-containing protein	*Nematostella vectensis*	2.2	Functions in the Golgi complex and cilia formation	[112]
A0A913XEK1	BRICHOS domain-containing protein	*Exaiptasia diaphana*	3.9	Regulates cell proliferation	[52]
**Variable temperature group**					
**Accession number**	**Identified protein**	**Organism**	**% coverage**	**Function**	**Reference**
A0A913YGN7	DED domain-containing protein	*Exaiptasia diaphana*	2.1	Essential in embryonic development and homeostasis of the immune system	[113]
A0A6P8IYN5	Cysteine and glycine-rich protein 1-like	*Actinia tenebrosa*	13	Gene transcription, cell movements, and cell proliferation	[48]
A7SSB7	G-protein coupled receptors family 1 profile domain-containing protein (Fragment)	*Nematostella vectensis*	2.5	Transduction of extracellular stimuli into intracellular signals	[63]
A0A6P8J3T8	Dentin sialophosphoprotein-like	*Exaiptasia diaphana*	0.32	Cell differentiation	[53]
A0A6P8J6M5	Calmodulin-like protein 12	*Actinia tenebrosa*	13	Controls diverse cellular functions	[61]

				by regulating the activity of various target proteins	
A0A6P8HR02	Claspin-like	*Actinia tenebrosa*	3.0	Cell homeostasis	[114]
A0A6P8HH99	Deleted in malignant brain tumors 1 protein-like	*Actinia tenebrosa*	1.5	Mucosal immune defense, epithelial differentiation, and tumor suppression	[115]
A7RP60	SSD domain-containing protein	*Nematostella vectensis*	1.9	Plays a key role in sterol sensing, transport, and signaling	[116]

References

(1) Stabili, L.; Pagliara, P. Effect of zinc on lysozyme-like activity of the seastar Marthasterias glacialis (Echinodermata, Asteroidea) mucus. *Journal of Invertebrate Pathology* **2009**, *100* (3), 189-192. DOI: https://doi.org/10.1016/j.jip.2009.01.005.
(2) Sun, Y.-X.; Chen, C.; Xu, W.-J.; Abbas, M. N.; Mu, F.-F.; Ding, W.-J.; Zhang, H.-J.; Li, J. Functions of Bombyx mori cathepsin L-like in innate immune response and anti-microbial autophagy. *Developmental & Comparative Immunology* **2021**, *116*, 103927. DOI: https://doi.org/10.1016/j.dci.2020.103927.
(3) Shortall, K.; Djeghader, A.; Magner, E.; Soulimane, T. Insights into Aldehyde Dehydrogenase Enzymes: A Structural Perspective. *Frontiers in Molecular Biosciences* **2021**, *8*, Review. DOI: 10.3389/fmolb.2021.659550.
(4) Chen, W.; Wang, X.; Li, X.; Lv, X.; Zhou, C.; Deng, C.; Lei, H.; Men, J.; Fan, Y.; Liang, C.; et al. Molecular characterization of cathepsin B from Clonorchis sinensis excretory/secretory products and assessment of its potential for serodiagnosis of clonorchiasis. *Parasites & Vectors* **2011**, *4* (1), 149. DOI: 10.1186/1756-3305-4-149.
(5) Neupane, P.; Bhuju, S.; Thapa, N.; Bhattarai, H. K. ATP Synthase: Structure, Function and Inhibition. *Biomolecular Concepts* **2019**, *10* (1), 1-10. DOI: doi:10.1515/bmc-2019-0001 (acccessed 2024-11-28).
(6) Kumar, S.; Sandell, L. L.; Trainor, P. A.; Koentgen, F.; Duester, G. Alcohol and aldehyde dehydrogenases: Retinoid metabolic effects in mouse knockout models. *Biochimica et Biophysica Acta (BBA) - Molecular and Cell Biology of Lipids* **2012**, *1821* (1), 198-205. DOI: https://doi.org/10.1016/j.bbalip.2011.04.004.
(7) Nandi, A.; Yan, L.-J.; Jana, C. K.; Das, N. Role of Catalase in Oxidative Stress- and Age-Associated Degenerative Diseases. *Oxidative Medicine and Cellular Longevity* **2019**, *2019* (1), 9613090. DOI: https://doi.org/10.1155/2019/9613090.
(8) Gavini, N.; Tungtur, S.; Pulakat, L. Peptidyl-Prolyl cis/trans Isomerase-Independent Functional NifH Mutant of Azotobacter vinelandii. *Journal of Bacteriology* **2006**, *188* (16), 6020-6025. DOI: doi:10.1128/jb.00379-06.
(9) Vizán, P.; Di Croce, L.; Aranda, S. Functional and Pathological Roles of AHCY. *Frontiers in Cell and Developmental Biology* **2021**, *9*, Mini Review. DOI: 10.3389/fcell.2021.654344.

(10) Mitrović, A.; Pečar Fonović, U.; Kos, J. Cysteine cathepsins B and X promote epithelial-mesenchymal transition of tumor cells. *European Journal of Cell Biology* **2017**, *96* (6), 622-631. DOI: https://doi.org/10.1016/j.ejcb.2017.04.003.
(11) Fukano, K.; Kimura, K. Chapter Six - Measurement of Enolase Activity in Cell Lysates. In *Methods in Enzymology*, Galluzzi, L., Kroemer, G. Eds.; Vol. 542; Academic Press, 2014; pp 115-124.
(12) Kim, J. T.; Li, V. L.; Terrell, S. M.; Fischer, C. R.; Long, J. Z. Family-wide Annotation of Enzymatic Pathways by Parallel In Vivo Metabolomics. *Cell Chemical Biology* **2019**, *26* (11), 1623-1629.e1623. DOI: https://doi.org/10.1016/j.chembiol.2019.09.009.
(13) Chan, S. J.; Oliva, A. A.; LaMendola, J.; Grens, A.; Bode, H.; Steiner, D. F. Conservation of the prohormone convertase gene family in metazoa: analysis of cDNAs encoding a PC3-like protein from hydra. *Proceedings of the National Academy of Sciences* **1992**, *89* (15), 6678-6682. DOI: doi:10.1073/pnas.89.15.6678.
(14) Laurindo, F. R. M.; Pescatore, L. A.; de Castro Fernandes, D. Protein disulfide isomerase in redox cell signaling and homeostasis. *Free Radical Biology and Medicine* **2012**, *52* (9), 1954-1969. DOI: https://doi.org/10.1016/j.freeradbiomed.2012.02.037.
(15) Shaw, P. E. Peptidyl-prolyl isomerases: a new twist to transcription. *EMBO reports* **2002**, *3* (6), 521-526. DOI: https://doi.org/10.1093/embo-reports/kvf118.
(16) Franco, S. J.; Huttenlocher, A. Regulating cell migration: calpains make the cut. *Journal of Cell Science* **2005**, *118* (17), 3829-3838. DOI: 10.1242/jcs.02562 (acccessed 11/28/2024).
(17) Tomassetti, M.; Garavaglia, B. S.; Vranych, C. V.; Gottig, N.; Ottado, J.; Gramajo, H.; Diacovich, L. 3-methylcrotonyl Coenzyme A (CoA) carboxylase complex is involved in the Xanthomonas citri subsp. citri lifestyle during citrus infection. *PLOS ONE* **2018**, *13* (6), e0198414. DOI: 10.1371/journal.pone.0198414.
(18) Qvit, N. Chapter 15 - Therapeutic peptides targeting protein kinase: progress, challenges, and future directions, featuring cancer and cardiovascular disease. In *Peptide and Peptidomimetic Therapeutics*, Qvit, N., Rubin, S. J. S. Eds.; Academic Press, 2022; pp 333-356.
(19) Schnell, J. R.; Dyson, H. J.; Wright, P. E. Structure, Dynamics, and Catalytic Function of Dihydrofolate Reductase. *Annual Review of Biophysics* **2004**, *33* (Volume 33, 2004), 119-140. DOI: https://doi.org/10.1146/annurev.biophys.33.110502.133613.
(20) Wilkinson, B.; Gilbert, H. F. Protein disulfide isomerase. *Biochimica et Biophysica Acta (BBA) - Proteins and Proteomics* **2004**, *1699* (1), 35-44. DOI: https://doi.org/10.1016/j.bbapap.2004.02.017.
(21) Son, H. F.; Kim, K.-J. Structural Insights into a Novel Class of Aspartate Aminotransferase from Corynebacterium glutamicum. *PLOS ONE* **2016**, *11* (6), e0158402. DOI: 10.1371/journal.pone.0158402.

(22) Lu, M.; Tian, X.; Tian, A.-L.; Li, C.; Yan, R.; Xu, L.; Song, X.; Li, X. A Novel α/β Hydrolase Domain Protein Derived From Haemonchus contortus Acts at the Parasite-Host Interface. *Frontiers in Immunology* **2020**, *11*, Original Research. DOI: 10.3389/fimmu.2020.01388.
(23) Hishinuma, E.; Narita, Y.; Saito, S.; Maekawa, M.; Akai, F.; Nakanishi, Y.; Yasuda, J.; Nagasaki, M.; Yamamoto, M.; Yamaguchi, H.; et al. Functional Characterization of 21 Allelic Variants of Dihydropyrimidine Dehydrogenase Identified in 1070 Japanese Individuals. *Drug Metabolism and Disposition* **2018**, *46* (8), 1083. DOI: 10.1124/dmd.118.081737.
(24) Boomgaarden, I.; Vock, C.; Klapper, M.; Döring, F. Comparative Analyses of Disease Risk Genes Belonging to the Acyl-CoA Synthetase Medium-Chain (ACSM) Family in Human Liver and Cell Lines. *Biochemical Genetics* **2009**, *47* (9), 739-748. DOI: 10.1007/s10528-009-9273-z.
(25) Banks, J. A.; Nishiyama, T.; Hasebe, M.; Bowman, J. L.; Gribskov, M.; dePamphilis, C.; Albert, V. A.; Aono, N.; Aoyama, T.; Ambrose, B. A.; et al. The Selaginella Genome Identifies Genetic Changes Associated with the Evolution of Vascular Plants. *Science* **2011**, *332* (6032), 960-963. DOI: doi:10.1126/science.1203810.
(26) Paukku, K.; Yang, J.; Silvennoinen, O. Tudor and Nuclease-Like Domains Containing Protein p100 Function as Coactivators for Signal Transducer and Activator of Transcription 5. *Molecular Endocrinology* **2003**, *17* (9), 1805-1814. DOI: 10.1210/me.2002-0256 (acccessed 11/28/2024).
(27) He, M.; Pei, Z.; Mohsen, A.-W.; Watkins, P.; Murdoch, G.; Van Veldhoven, P. P.; Ensenauer, R.; Vockley, J. Identification and characterization of new long chain Acyl-CoA dehydrogenases. *Molecular Genetics and Metabolism* **2011**, *102* (4), 418-429. DOI: https://doi.org/10.1016/j.ymgme.2010.12.005.
(28) Caswell, B. T.; de Carvalho, C. C.; Nguyen, H.; Roy, M.; Nguyen, T.; Cantu, D. C. Thioesterase enzyme families: Functions, structures, and mechanisms. *Protein Science* **2022**, *31* (3), 652-676. DOI: https://doi.org/10.1002/pro.4263.
(29) Abe, A.; Shayman, J. A. Sphingolipid Catabolism. In *Encyclopedia of Biological Chemistry (Second Edition)*, Lennarz, W. J., Lane, M. D. Eds.; Academic Press, 2013; pp 287-292.
(30) Rivieccio, M. A.; Suh, H.-S.; Zhao, Y.; Zhao, M.-L.; Chin, K. C.; Lee, S. C.; Brosnan, C. F. TLR3 Ligation Activates an Antiviral Response in Human Fetal Astrocytes: A Role for Viperin/cig51. *The Journal of Immunology* **2006**, *177* (7), 4735-4741. DOI: 10.4049/jimmunol.177.7.4735 (acccessed 11/28/2024).
(31) Hirano, T.; Matsuzawa, T.; Takegawa, K.; Sato, M. H. Loss-of-Function and Gain-of-Function Mutations in FAB1A/B Impair Endomembrane Homeostasis, Conferring Pleiotropic Developmental Abnormalities in Arabidopsis *Plant Physiology* **2010**, *155* (2), 797-807. DOI: 10.1104/pp.110.167981 (acccessed 11/28/2024).

(32) Gonzalez, C. F.; Proudfoot, M.; Brown, G.; Korniyenko, Y.; Mori, H.; Savchenko, A. V.; Yakunin, A. F. Molecular Basis of Formaldehyde Detoxification: CHARACTERIZATION OF TWO S-FORMYLGLUTATHIONE HYDROLASES FROM ESCHERICHIA COLI, FrmB AND YeiG *Journal of Biological Chemistry* **2006**, *281* (20), 14514-14522. DOI: 10.1074/jbc.M600996200 (acccessed 2024/11/27).
(33) Devi, S. S. 9.02 - Structure and Function of Hepatic Parenchymal Cells*. In *Comprehensive Toxicology (Second Edition)*, McQueen, C. A. Ed.; Elsevier, 2010; pp 11-30.
(34) Hung, G.-C.; Brown, C. R.; Wolfe, A. B.; Liu, J.; Chiang, H.-L. Degradation of the Gluconeogenic Enzymes Fructose-1,6-bisphosphatase and Malate Dehydrogenase Is Mediated by Distinct Proteolytic Pathways and Signaling Events *. *Journal of Biological Chemistry* **2004**, *279* (47), 49138-49150. DOI: 10.1074/jbc.M404544200 (acccessed 2024/11/27).
(35) Fulcher, L. J.; Sapkota, G. P. Functions and regulation of the serine/threonine protein kinase CK1 family: moving beyond promiscuity. *Biochemical Journal* **2020**, *477* (23), 4603-4621. DOI: 10.1042/bcj20200506 (acccessed 11/28/2024).
(36) Joazeiro, C. A. P.; Weissman, A. M. RING Finger Proteins: Mediators of Ubiquitin Ligase Activity. *Cell* **2000**, *102* (5), 549-552. DOI: 10.1016/S0092-8674(00)00077-5 (acccessed 2024/11/27).
(37) Schneider, T.; Martinez-Martinez, A.; Cubillos-Rojas, M.; Bartrons, R.; Ventura, F.; Rosa, J. L. The E3 ubiquitin ligase HERC1 controls the ERK signaling pathway targeting C-RAF for degradation. *Oncotarget* **2018**, *9* (59), 31531-31548. DOI: 10.18632/oncotarget.25847 From NLM.
(38) Paul, D.; Bargale, A. B.; Rapole, S.; Shetty, P. K.; Santra, M. K. Protein Phosphatase 1 Regulatory Subunit SDS22 Inhibits Breast Cancer Cell Tumorigenesis by Functioning as a Negative Regulator of the AKT Signaling Pathway. *Neoplasia* **2019**, *21* (1), 30-40. DOI: https://doi.org/10.1016/j.neo.2018.10.009.
(39) Kohlmeier, M. Chapter 8 - Amino Acids and Nitrogen Compounds. In *Nutrient Metabolism (Second Edition)*, Kohlmeier, M. Ed.; Academic Press, 2015; pp 265-477.
(40) Taylor, A. Aminopeptidases: structure and function. *The FASEB Journal* **1993**, *7* (2), 290-298. DOI: https://doi.org/10.1096/fasebj.7.2.8440407.
(41) McMullen, B. A.; Fujikawa, K.; Davie, E. W. Location of the disulfide bonds in human plasma prekallikrein: the presence of four novel apple domains in the amino-terminal portion of the molecule. *Biochemistry* **1991**, *30* (8), 2050-2056. DOI: 10.1021/bi00222a007.
(42) Cantor, J. R.; Stone, E. M.; Chantranupong, L.; Georgiou, G. The Human Asparaginase-like Protein 1 hASRGL1 Is an Ntn Hydrolase with β-Aspartyl Peptidase Activity. *Biochemistry* **2009**, *48* (46), 11026-11031. DOI: 10.1021/bi901397h.
(43) Otten, E. G.; Werner, E.; Crespillo-Casado, A.; Boyle, K. B.; Dharamdasani, V.; Pathe, C.; Santhanam, B.; Randow, F. Ubiquitylation of lipopolysaccharide by RNF213

during bacterial infection. *Nature* **2021**, *594* (7861), 111-116. DOI: 10.1038/s41586-021-03566-4.
(44) Allocati, N.; Masulli, M.; Di Ilio, C.; Federici, L. Glutathione transferases: substrates, inihibitors and pro-drugs in cancer and neurodegenerative diseases. *Oncogenesis* **2018**, *7* (1), 8. DOI: 10.1038/s41389-017-0025-3.
(45) Akoh, C. C.; Lee, G.-C.; Liaw, Y.-C.; Huang, T.-H.; Shaw, J.-F. GDSL family of serine esterases/lipases. *Progress in Lipid Research* **2004**, *43* (6), 534-552. DOI: https://doi.org/10.1016/j.plipres.2004.09.002.
(46) Yang, R.-Z.; Park, S.; Reagan, W. J.; Goldstein, R.; Zhong, S.; Lawton, M.; Rajamohan, F.; Qian, K.; Liu, L.; Gong, D.-W. Alanine aminotransferase isoenzymes: Molecular cloning and quantitative analysis of tissue expression in rats and serum elevation in liver toxicity# %U https://journals.lww.com/hep/fulltext/2009/02000/alanine_aminotransferase_isoenzymes__molecular.32.aspx. *Hepatology* **2009**, *49* (2), 598-607. DOI: 10.1002/hep.22657.
(47) Dores-Silva, P. R.; Cauvi, D. M.; Coto, A. L. S.; Silva, N. S. M.; Borges, J. C.; De Maio, A. Human heat shock cognate protein (HSC70/HSPA8) interacts with negatively charged phospholipids by a different mechanism than other HSP70s and brings HSP90 into membranes. *Cell Stress and Chaperones* **2021**, *26* (4), 671-684. DOI: https://doi.org/10.1007/s12192-021-01210-8.
(48) Ma, L.; Yu, Y.-M.; Guo, Y.; Hart, R. P.; Schachner, M. Cysteine- and glycine-rich protein 1a is involved in spinal cord regeneration in adult zebrafish. *European Journal of Neuroscience* **2012**, *35* (3), 353-365. DOI: https://doi.org/10.1111/j.1460-9568.2011.07958.x (acccessed 2024/11/28).
(49) Pobre, K. F. R.; Poet, G. J.; Hendershot, L. M. The endoplasmic reticulum (ER) chaperone BiP is a master regulator of ER functions: Getting by with a little help from ERdj friends. *Journal of Biological Chemistry* **2019**, *294* (6), 2098-2108. DOI: 10.1074/jbc.REV118.002804 (acccessed 2024/11/28).
(50) Terry, A.; de Natale Rosario, E.; Politis, M. Chapter 19 - Neuroimaging in dystonia. In *Neuroimaging in Parkinson's Disease and Related Disorders*, Politis, M., Wilson, H., De Natale, E. R. Eds.; Academic Press, 2023; pp 541-566.
(51) Bastien, B. L.; Cowen, M. H.; Hart, M. P. Distinct neurexin isoforms cooperate to initiate and maintain foraging activity. *Translational Psychiatry* **2023**, *13* (1), 367. DOI: 10.1038/s41398-023-02668-z.
(52) Rolland, T.; Taşan, M.; Charloteaux, B.; Pevzner, Samuel J.; Zhong, Q.; Sahni, N.; Yi, S.; Lemmens, I.; Fontanillo, C.; Mosca, R.; et al. A Proteome-Scale Map of the Human Interactome Network. *Cell* **2014**, *159* (5), 1212-1226. DOI: 10.1016/j.cell.2014.10.050 (acccessed 2024/11/28).
(53) Ritchie, H. The functional significance of dentin sialoprotein-phosphophoryn and dentin sialoprotein. *International Journal of Oral Science* **2018**, *10* (4), 31. DOI: 10.1038/s41368-018-0035-9.

(54) Serrano, M.; Lin, A. W.; McCurrach, M. E.; Beach, D.; Lowe, S. W. Oncogenic Provokes Premature Cell Senescence Associated with Accumulation of p53 and p16. *Cell* **1997**, *88* (5), 593-602. DOI: 10.1016/S0092-8674(00)81902-9 (acccessed 2024/11/28).
(55) Yang, D.; Zhou, Q.; Labroska, V.; Qin, S.; Darbalaei, S.; Wu, Y.; Yuliantie, E.; Xie, L.; Tao, H.; Cheng, J.; et al. G protein-coupled receptors: structure- and function-based drug discovery. *Signal Transduction and Targeted Therapy* **2021**, *6* (1), 7. DOI: 10.1038/s41392-020-00435-w.
(56) Recio, C.; Guerra, B.; Guerra-Rodríguez, M.; Aranda-Tavío, H.; Martín-Rodríguez, P.; de Mirecki-Garrido, M.; Brito-Casillas, Y.; García-Castellano, J. M.; Estévez-Braun, A.; Fernández-Pérez, L. Signal transducer and activator of transcription (STAT)-5: an opportunity for drug development in oncohematology. *Oncogene* **2019**, *38* (24), 4657-4668. DOI: 10.1038/s41388-019-0752-3.
(57) Boulter, E.; Garcia-Mata, R.; Guilluy, C.; Dubash, A.; Rossi, G.; Brennwald, P. J.; Burridge, K. Regulation of Rho GTPase crosstalk, degradation and activity by RhoGDI1. *Nature Cell Biology* **2010**, *12* (5), 477-483. DOI: 10.1038/ncb2049.
(58) Philippe, R.; Antigny, F.; Buscaglia, P.; Norez, C.; Huguet, F.; Castelbou, C.; Trouvé, P.; Becq, F.; Frieden, M.; Férec, C.; et al. Calumenin contributes to ER-Ca2+ homeostasis in bronchial epithelial cells expressing WT and F508del mutated CFTR and to F508del-CFTR retention. *Cell Calcium* **2017**, *62*, 47-59. DOI: https://doi.org/10.1016/j.ceca.2017.01.011.
(59) Nisemblat, S.; Yaniv, O.; Parnas, A.; Frolow, F.; Azem, A. Crystal structure of the human mitochondrial chaperonin symmetrical football complex. *Proceedings of the National Academy of Sciences* **2015**, *112* (19), 6044-6049. DOI: 10.1073/pnas.1411718112 (acccessed 2024/11/28).
(60) Cappadocia, L.; Lima, C. D. Ubiquitin-like Protein Conjugation: Structures, Chemistry, and Mechanism. *Chemical Reviews* **2018**, *118* (3), 889-918. DOI: 10.1021/acs.chemrev.6b00737.
(61) Zhang, J.; Zou, A.; Wen, Y.; Wei, X.; Liu, C.; Lv, X.; Ma, X.; Fan, G.; Sun, X. SlCML55, a novel Solanum lycopersicum calmodulin-like gene, negatively regulates plant immunity to Phytophthora pathogens. *Scientia Horticulturae* **2022**, *299*, 111049. DOI: https://doi.org/10.1016/j.scienta.2022.111049.
(62) Lu, Y.-C.; Weng, W.-C.; Lee, H. Functional Roles of Calreticulin in Cancer Biology. *BioMed Research International* **2015**, *2015* (1), 526524. DOI: https://doi.org/10.1155/2015/526524 (acccessed 2024/11/28).
(63) Symonds, K.; Teresinski, H. J.; Hau, B.; Dwivedi, V.; Belausov, E.; Bar-Sinai, S.; Tominaga, M.; Haraguchi, T.; Sadot, E.; Ito, K.; et al. Functional characterization of calmodulin-like proteins, CML13 and CML14, as novel light chains of Arabidopsis class VIII myosins. *Journal of Experimental Botany* **2024**, *75* (8), 2313-2329. DOI: 10.1093/jxb/erae031 (acccessed 11/29/2024).
(64) Civiero, L.; Dihanich, S.; Lewis, Patrick A.; Greggio, E. Genetic, Structural, and Molecular Insights into the Function of Ras of Complex Proteins Domains. *Chemistry &*

Biology **2014**, *21* (7), 809-818. DOI: 10.1016/j.chembiol.2014.05.010 (acccessed 2024/11/28).
(65) Flores-Benitez, D.; Knust, E. Dynamics of epithelial cell polarity in Drosophila: how to regulate the regulators? *Current Opinion in Cell Biology* **2016**, *42*, 13-21. DOI: https://doi.org/10.1016/j.ceb.2016.03.018.
(66) Turanov, A. A.; Hatfield, D. L.; Gladyshev, V. N. Chapter 14 - Characterization of Protein Targets of Mammalian Thioredoxin Reductases. In *Methods in Enzymology*, Cadenas, E., Packer, L. Eds.; Vol. 474; Academic Press, 2010; pp 245-254.
(67) Hoeflich, K. P.; Ikura, M. Radixin: cytoskeletal adopter and signaling protein. *The International Journal of Biochemistry & Cell Biology* **2004**, *36* (11), 2131-2136. DOI: https://doi.org/10.1016/j.biocel.2003.11.018.
(68) Hong, W.; Guan, K.-L. The YAP and TAZ transcription co-activators: Key downstream effectors of the mammalian Hippo pathway. *Seminars in Cell & Developmental Biology* **2012**, *23* (7), 785-793. DOI: https://doi.org/10.1016/j.semcdb.2012.05.004.
(69) Schimanski, C. C.; Schmitz, G.; Kashyap, A.; Bosserhoff, A. K.; Bataille, F.; Schäfer, S. C.; Lehr, H. A.; Berger, M. R.; Galle, P. R.; Strand, S.; et al. Reduced expression of Hugl-1, the human homologue of Drosophila tumour suppressor gene lgl, contributes to progression of colorectal cancer. *Oncogene* **2005**, *24* (19), 3100-3109. DOI: 10.1038/sj.onc.1208520.
(70) Xiao, J.; Liu, C.-C.; Chen, P.-L.; Lee, W.-H. RINT-1, a Novel Rad50-interacting Protein, Participates in Radiation-induced G2/M Checkpoint Control *Journal of Biological Chemistry* **2001**, *276* (9), 6105-6111. DOI: 10.1074/jbc.M008893200 (acccessed 2024/11/28).
(71) Wong, S.-W.; Sil, P.; Martinez, J. Rubicon: LC3-associated phagocytosis and beyond. *The FEBS Journal* **2018**, *285* (8), 1379-1388. DOI: https://doi.org/10.1111/febs.14354 (acccessed 2024/11/28).
(72) Julius, M. A.; Schelbert, B.; Hsu, W.; Fitzpatrick, E.; Jho, E.; Fagotto, F.; Costantini, F.; Kitajewski, J. Domains of Axin and Disheveled Required for Interaction and Function in Wnt Signaling. *Biochemical and Biophysical Research Communications* **2000**, *276* (3), 1162-1169. DOI: https://doi.org/10.1006/bbrc.2000.3607.
(73) Zhang, Y.; Shen, H.; Liu, H.; Feng, H.; Liu, Y.; Zhu, X.; Liu, X. Arp2/3 complex controls T cell homeostasis by maintaining surface TCR levels via regulating TCR+ endosome trafficking. *Scientific Reports* **2017**, *7* (1), 8952. DOI: 10.1038/s41598-017-08357-4.
(74) Ishizaki, H.; Miyoshi, J.; Kamiya, H.; Togawa, A.; Tanaka, M.; Sasaki, T.; Endo, K.; Mizoguchi, A.; Ozawa, S.; Takai, Y. Role of Rab GDP dissociation inhibitor α in regulating plasticity of hippocampal neurotransmission. *Proceedings of the National Academy of Sciences* **2000**, *97* (21), 11587-11592. DOI: 10.1073/pnas.97.21.11587 (acccessed 2024/11/28).

(75) Forbes, J. G.; Flaherty, D. B.; Ma, K.; Qadota, H.; Benian, G. M.; Wang, K. Extensive and Modular Intrinsically Disordered Segments in C. elegans TTN-1 and Implications in Filament Binding, Elasticity and Oblique Striation. *Journal of Molecular Biology* **2010**, *398* (5), 672-689. DOI: https://doi.org/10.1016/j.jmb.2010.03.032.
(76) Boura, E.; Nencka, R. Phosphatidylinositol 4-kinases: Function, structure, and inhibition. *Experimental Cell Research* **2015**, *337* (2), 136-145. DOI: https://doi.org/10.1016/j.yexcr.2015.03.028.
(77) Yu, J. E.; Yeo, I. J.; Han, S.-B.; Yun, J.; Kim, B.; Yong, Y. J.; Lim, Y.-s.; Kim, T. H.; Son, D. J.; Hong, J. T. Significance of chitinase-3-like protein 1 in the pathogenesis of inflammatory diseases and cancer. *Experimental & Molecular Medicine* **2024**, *56* (1), 1-18. DOI: 10.1038/s12276-023-01131-9.
(78) Butterfield, D. A.; Hardas, S. S.; Lange, M. L. B. Oxidatively Modified Glyceraldehyde-3-Phosphate Dehydrogenase (GAPDH) and Alzheimer's Disease: Many Pathways to Neurodegeneration. *Journal of Alzheimer's Disease* **2010**, *20*, 369-393. DOI: 10.3233/JAD-2010-1375.
(79) Lee, C. G.; Da Silva, C. A.; Dela Cruz, C. S.; Ahangari, F.; Ma, B.; Kang, M.-J.; He, C.-H.; Takyar, S.; Elias, J. A. Role of Chitin and Chitinase/Chitinase-Like Proteins in Inflammation, Tissue Remodeling, and Injury. *Annual Review of Physiology* **2011**, *73* (Volume 73, 2011), 479-501. DOI: https://doi.org/10.1146/annurev-physiol-012110-142250.
(80) Ternes, P.; Franke, S.; Zähringer, U.; Sperling, P.; Heinz, E. Identification and Characterization of a Sphingolipid Δ4-Desaturase Family *Journal of Biological Chemistry* **2002**, *277* (28), 25512-25518. DOI: 10.1074/jbc.M202947200 (acccessed 2024/11/28).
(81) Patnala, H. S.; Kabilan, U.; Gopalakrishnan, L.; Rao, R. M. D.; Kumar, D. S. Chapter Four - Marine Fungal and Bacterial Isolates for Lipase Production: A Comparative Study. In *Advances in Food and Nutrition Research*, Kim, S.-K., Toldrá, F. Eds.; Vol. 78; Academic Press, 2016; pp 71-94.
(82) Cooke, F. T.; Dove, S. K.; McEwen, R. K.; Painter, G.; Holmes, A. B.; Hall, M. N.; Michell, R. H.; Parker, P. J. The stress-activated phosphatidylinositol 3-phosphate 5-kinase Fab1p is essential for vacuole function in S. cerevisiae. *Current Biology* **1998**, *8* (22), S1-S2. DOI: 10.1016/S0960-9822(07)00513-1 (acccessed 2024/11/28).
(83) Rawlings, N. D.; Barrett, A. J. [2] Families of serine peptidases. In *Methods in Enzymology*, Vol. 244; Academic Press, 1994; pp 19-61.
(84) Biederbick, A.; Kosan, C.; Kunz, J.; Elsässer, H.-P. First Apyrase Splice Variants Have Different Enzymatic Properties *. *Journal of Biological Chemistry* **2000**, *275* (25), 19018-19024. DOI: 10.1074/jbc.M001245200 (acccessed 2024/11/28).
(85) Gingras, R.; Richard, C.; El-Alfy, M.; Morales, C. R.; Potier, M.; Pshezhetsky, A. V. Purification, cDNA Cloning, and Expression of a New Human Blood Plasma Glutamate Carboxypeptidase Homologous to N-Acetyl-aspartyl-1;-glutamate

Carboxypeptidase/Prostate-specific Membrane Antigen *Journal of Biological Chemistry* **1999**, *274* (17), 11742-11750. DOI: 10.1074/jbc.274.17.11742 (acccessed 2024/11/28).
(86) Clark, H. F.; Gurney, A. L.; Abaya, E.; Baker, K.; Baldwin, D.; Brush, J.; Chen, J.; Chow, B.; Chui, C.; Crowley, C.; et al. The Secreted Protein Discovery Initiative (SPDI), a Large-Scale Effort to Identify Novel Human Secreted and Transmembrane Proteins: A Bioinformatics Assessment. *Genome Research* **2003**, *13* (10), 2265-2270. DOI: 10.1101/gr.1293003.
(87) Pey, A. L.; Albert, A.; Salido, E. Protein Homeostasis Defects of Alanine-Glyoxylate Aminotransferase: New Therapeutic Strategies in Primary Hyperoxaluria Type I. *BioMed Research International* **2013**, *2013* (1), 687658. DOI: https://doi.org/10.1155/2013/687658.
(88) Pratt, A. C.; Dewage, S. W.; Pang, A. H.; Biswas, T.; Barnard-Britson, S.; Cisneros, G. A.; Tsodikov, O. V. Structural and computational dissection of the catalytic mechanism of the inorganic pyrophosphatase from Mycobacterium tuberculosis. *Journal of Structural Biology* **2015**, *192* (1), 76-87. DOI: https://doi.org/10.1016/j.jsb.2015.08.010.
(89) Tang, W.; Ji, Q.; Huang, Y.; Jiang, Z.; Bao, M.; Wang, H.; Lin, R. FAR-RED ELONGATED HYPOCOTYL3 and FAR-RED IMPAIRED RESPONSE1 Transcription Factors Integrate Light and Abscisic Acid Signaling in Arabidopsis *Plant Physiology* **2013**, *163* (2), 857-866. DOI: 10.1104/pp.113.224386 (acccessed 11/29/2024).
(90) Huang, Q.-S.; Xie, X.-L.; Liang, G.; Gong, F.; Wang, Y.; Wei, X.-Q.; Wang, Q.; Ji, Z.-L.; Chen, Q.-X. The GH18 family of chitinases: Their domain architectures, functions and evolutions. *Glycobiology* **2011**, *22* (1), 23-34. DOI: 10.1093/glycob/cwr092 (acccessed 11/29/2024).
(91) Bardal, S. K.; Waechter, J. E.; Martin, D. S. Chapter 18 - Infectious Diseases. In *Applied Pharmacology*, Bardal, S. K., Waechter, J. E., Martin, D. S. Eds.; W.B. Saunders, 2011; pp 233-291.
(92) Qiu, X.; Xie, X.; Meesapyodsuk, D. Molecular mechanisms for biosynthesis and assembly of nutritionally important very long chain polyunsaturated fatty acids in microorganisms. *Progress in Lipid Research* **2020**, *79*, 101047. DOI: https://doi.org/10.1016/j.plipres.2020.101047.
(93) Haneda, M.; Sugimoto, T.; Kikkawa, R. Mitogen-activated protein kinase phosphatase: a negative regulator of the mitogen-activated protein kinase cascade. *European Journal of Pharmacology* **1999**, *365* (1), 1-7. DOI: https://doi.org/10.1016/S0014-2999(98)00857-7.
(94) Niemi, N. M.; Sacoman, J. L.; Westrate, L. M.; Gaither, L. A.; Lanning, N. J.; Martin, K. R.; MacKeigan, J. P. The Pseudophosphatase MK-STYX Physically and Genetically Interacts with the Mitochondrial Phosphatase PTPMT1. *PLOS ONE* **2014**, *9* (4), e93896. DOI: 10.1371/journal.pone.0093896.
(95) Luo, S.; Tong, L. Structure and Function of the Separase-Securin Complex. In *Macromolecular Protein Complexes III: Structure and Function*, Harris, J. R., Marles-Wright, J. Eds.; Springer International Publishing, 2021; pp 217-232.

(96) Fonović, U. P.; Mitrović, A.; Knez, D.; Jakoš, T.; Pišlar, A.; Brus, B.; Doljak, B.; Stojan, J.; Žakelj, S.; Trontelj, J.; et al. Identification and characterization of the novel reversible and selective cathepsin X inhibitors. *Scientific Reports* **2017**, *7* (1), 11459. DOI: 10.1038/s41598-017-11935-1.
(97) Lijun Lin, M.; Perryman, B.; Friedman, D.; Roberts, R.; Ma, T. S. Determination of the catalytic site of creatine kinase by site-directed mutagenesis. *Biochimica et Biophysica Acta (BBA) - Protein Structure and Molecular Enzymology* **1994**, *1206* (1), 97-104. DOI: https://doi.org/10.1016/0167-4838(94)90077-9.
(98) Hashimshony, T.; Feder, M.; Levin, M.; Hall, B. K.; Yanai, I. Spatiotemporal transcriptomics reveals the evolutionary history of the endoderm germ layer. *Nature* **2015**, *519* (7542), 219-222. DOI: 10.1038/nature13996.
(99) Kim, J.; Lee, J. E.; Heynen-Genel, S.; Suyama, E.; Ono, K.; Lee, K.; Ideker, T.; Aza-Blanc, P.; Gleeson, J. G. Functional genomic screen for modulators of ciliogenesis and cilium length. *Nature* **2010**, *464* (7291), 1048-1051. DOI: 10.1038/nature08895.
(100) Diggle, Christine P.; Sukoff Rizzo, Stacey J.; Popiolek, M.; Hinttala, R.; Schülke, J.-P.; Kurian, Manju A.; Carr, Ian M.; Markham, Alexander F.; Bonthron, David T.; Watson, C.; et al. Biallelic Mutations in PDE10A Lead to Loss of Striatal PDE10A and a Hyperkinetic Movement Disorder with Onset in Infancy. *The American Journal of Human Genetics* **2016**, *98* (4), 735-743. DOI: 10.1016/j.ajhg.2016.03.015 (acccessed 2024/11/28).
(101) Kumar, R.; Yang, J.; Eddy, R. L.; Byers, M. G.; Shows, T. B.; Stanley, P. Cloning and expression of the murine gene and chromosomal location of the human gene encoding N-acetylglucosaminyltransferase I. *Glycobiology* **1992**, *2* (4), 383-393. DOI: 10.1093/glycob/2.4.383 (acccessed 11/29/2024).
(102) Komine, Y.; Kitabatake, M.; Yokogawa, T.; Nishikawa, K.; Inokuchi, H. A tRNA-like structure is present in 10Sa RNA, a small stable RNA from Escherichia coli. *Proceedings of the National Academy of Sciences* **1994**, *91* (20), 9223-9227. DOI: 10.1073/pnas.91.20.9223 (acccessed 2024/11/28).
(103) Downs, M.; Johnson, P.; Zeece, M. Chapter 9 - Insects and Their Connection to Food Allergy. In *Insects as Sustainable Food Ingredients*, Dossey, A. T., Morales-Ramos, J. A., Rojas, M. G. Eds.; Academic Press, 2016; pp 255-272.
(104) Polticelli, F.; Basran, J.; Faso, C.; Cona, A.; Minervini, G.; Angelini, R.; Federico, R.; Scrutton, N. S.; Tavladoraki, P. Lys300 Plays a Major Role in the Catalytic Mechanism of Maize Polyamine Oxidase. *Biochemistry* **2005**, *44* (49), 16108-16120. DOI: 10.1021/bi050983i.
(105) Aluganti Narasimhulu, C.; Singla, D. K. The Role of Bone Morphogenetic Protein 7 (BMP-7) in Inflammation in Heart Diseases. In *Cells*, 2020; Vol. 9.
(106) Han, S.; Lear, T. B.; Jerome, J. A.; Rajbhandari, S.; Snavely, C. A.; Gulick, D. L.; Gibson, K. F.; Zou, C.; Chen, B. B.; Mallampalli, R. K. Lipopolysaccharide Primes the NALP3 Inflammasome by Inhibiting Its Ubiquitination and Degradation Mediated by the

SCF^{FBXL2} E3 Ligase *. *Journal of Biological Chemistry* **2015**, *290* (29), 18124-18133. DOI: 10.1074/jbc.M115.645549 (acccessed 2024/11/28).

(107) Ouyang, C.; Mu, J.; Lu, Q.; Li, J.; Zhu, H.; Wang, Q.; Zou, M.-H.; Xie, Z. Autophagic degradation of KAT2A/GCN5 promotes directional migration of vascular smooth muscle cells by reducing TUBA/α-tubulin acetylation. *Autophagy* **2020**, *16* (10), 1753-1770. DOI: 10.1080/15548627.2019.1707488.

(108) Conte, C.; Griffis, E. R.; Hickson, I.; Perez-Oliva, A. B. USP45 and Spindly are part of the same complex implicated in cell migration. *Scientific Reports* **2018**, *8* (1), 14375. DOI: 10.1038/s41598-018-32685-8.

(109) Spencer, M. L.; Theodosiou, M.; Noonan, D. J. NPDC-1, a Novel Regulator of Neuronal Proliferation, Is Degraded by the Ubiquitin/Proteasome System through a PEST Degradation Motif *. *Journal of Biological Chemistry* **2004**, *279* (35), 37069-37078. DOI: 10.1074/jbc.M402507200 (acccessed 2024/11/28).

(110) Gaidt, M. M.; Morrow, A.; Fairgrieve, M. R.; Karr, J. P.; Yosef, N.; Vance, R. E. Self-guarding of MORC3 enables virulence factor-triggered immunity. *Nature* **2021**, *600* (7887), 138-142. DOI: 10.1038/s41586-021-04054-5.

(111) Shatov, V. M.; Gusev, N. B. Physico-chemical properties of two point mutants of small heat shock protein HspB6 (Hsp20) with abrogated cardioprotection. *Biochimie* **2020**, *174*, 126-135. DOI: https://doi.org/10.1016/j.biochi.2020.04.021.

(112) Donaldson, J. G.; Jackson, C. L. ARF family G proteins and their regulators: roles in membrane transport, development and disease. *Nature Reviews Molecular Cell Biology* **2011**, *12* (6), 362-375. DOI: 10.1038/nrm3117.

(113) Valmiki, M. G.; Ramos, J. W. Death Effector Domain-Containing Proteins. *Cellular and Molecular Life Sciences* **2009**, *66* (5), 814-830. DOI: 10.1007/s00018-008-8489-0.

(114) Azenha, D.; Lopes, M. C.; Martins, T. C. Claspin functions in cell homeostasis—A link to cancer? *DNA Repair* **2017**, *59*, 27-33. DOI: https://doi.org/10.1016/j.dnarep.2017.09.002.

(115) Park, H. S.; Kim, B. C.; Yeo, H. Y.; Kim, K.-H.; Yoo, B. C.; Park, J. W.; Chang, H. J. Deleted in malignant brain tumor 1 is a novel prognostic marker in colorectal cancer. *Oncol Rep* **2018**, *39* (5), 2279-2287. DOI: 10.3892/or.2018.6287.

(116) Wu, X.; Yan, R.; Cao, P.; Qian, H.; Yan, N. Structural advances in sterol-sensing domain-containing proteins. *Trends in Biochemical Sciences* **2022**, *47* (4), 289-300. DOI: 10.1016/j.tibs.2021.12.005.
